# Report on the Progress of Anatomy and Physiology in the Year 1842-3

**Published:** 1844-01

**Authors:** James Paget

**Affiliations:** Lecturer on General and Morbid Anatomy and Physiology, and Warden of the Collegiate Establishment at St. Bartholomew's Hospital


### REPORT ON THE PROGRESS OF ANATOMY AND PHYSIOLOGY IN THE YEAR 1842-3.

BY JAMES PAGET,
Lecturer on General and Morbid Anatomy and Physiology, and Warden of the Collegiate Establishment, at St. Bartholomew's Hospital.

THE general rule followed in this Report is that those works alone are noticed which were published between the first day of October, 1842, and the last of September, 1843. The author does not pretend to give an account of more than those new things which have, in his opinion, been proved or rendered probable in the period and in the department which the report embraces; nor is it pretended, after all the labour that has been bestowed upon it, that the account of these things is absolutely complete. Some works of importance have probably been inaccessible, others may have been overlooked or wrongly estimated; and therefore all those who find herein no notice of their recent physiological labours, have liberty to believe that the omission is the author's fault, not their's.

The arrangement of the subjects which the author has adopted is that of his own lectures; it is the one most convenient to himself, and probably will suit the purposes of as many readers as any other would.

### BLOOD.

Concerning the *general constitution* of the blood, MM. Andral and Gavarret, continuing with M. Delafond the observations already made on human blood, have examined that of many domestic animals, and have drawn these conclusions:* 1. In the species examined, the principal constituents of the blood are the same, but the proportions of each vary. 2. The highest natural average quantity of fibrin is in the herbivora, the lowest in the carnivora: the energy of constitution has no constant influence on the increase of the proportion of fibrin. 3. Of the corpuscles the highest average proportions are found in the carnivora, the lowest in the herbivora; and in the same species there is always an increase proportionate to the energy of the constitution; and in sheep, to the improvement of the breed. 4. During the first day after birth the fibrin is in very small quantity; the corpuscles comparatively abundant. 5. During the last periods of gestation, both fibrin and corpuscles fall below the healthy proportion; after parturition they increase to more than that proportion. 6. The fibrin (in the domestic animals as well as in man) is always increased in the inflammatory state: the corpuscles are never directly influenced in it. 7. The water of the blood has its lowest average in the carnivora, its highest in the herbivora. 8. Dropsy does not supervene on alteration of the blood, unless from diminution of albumen; excess of water or decrease of corpuscles will not produce it.

*Coagulation.* Dr. Polli,† from a long series of experiments on the influence of various gases on the coagulation of the blood, has shown how the discrepancy of previous experiments has depended on inattention to their details. All other con-

* Annales de Chimie et de Physique, 1842.
† Gazzetta Medica di Milano, Aprile 15, 1843.

ditions being the same, important differences as to time and mode of coagulation result from temporary exposure of the blood to the air, or from air remaining in the vessel, &c. Avoiding all these sources of fallacy, he found that coagulation takes place in pure oxygen or nitrogen, just as in atmospheric air; but that the presence of carbonic acid always impedes coagulation. Carbonic acid is always given off in coagulation ; and the greater the freedom with which it can be evolved, the more are the circumstances favorable to coagulation. The more carbonic acid the blood itself contains the slower is the coagulation, and the greater the chance of a buffy coat being formed; and a buffy coat being formed, without froth and over a dark clot, is always a sign of the blood being surcharged with carbonic acid.

*Buffy coat.* The exact mode of the formation of the buffy coat has been well illustrated by Mr. Wharton Jones,* whose observations on the blood I can in nearly all points confirm. He ascribes it, as Nasse and others did, chiefly to the tendency of the blood-corpuscles to arrange themselves rapidly in rolls (like rolls of coins,) which form a wide-meshed network, (as seen in a single layer under the microscope,) or a kind of spongework when they are kept in mass. In the former case the liquor sanguinis coagulates within the meshes of the rows of the corpuscles, and hence the distinctly mottled aspect of the layer when coagulated as well as when first drawn; in the latter case the spongework formed by the rows of corpuscles contracts and squeezes out the liquor sanguinis, and permits the greater specific gravity of the corpuscles to come into play, so that they sink quickly and the liquor sanguinis floats to the top and coagulates in a distinct layer. In both cases the pale corpuscles remain with the separated liquor sanguinis and are imbedded in its white coagulum.† The attraction for each other by which the corpuscles tend to unite in rolls, is so remarkably increased in the state of the blood in which a buffy coat is formed, that the early or instantaneous occurrence of this arrangement of them, as seen by the microscope in a single drop of blood, affords all the evidence which could be derived from the formation of a buffy coat on a large quantity.

*Corpuscles.* On the size of the blood-corpuscles, Mr. Gulliver has added to his former copious observations the measurements of those of many more species of mammalia and birds;‡ and on their structure he has adduced,§ from a comparison of the effects of water, acetic and muriatic acids, and other agents upon the blood-corpuscles of mammalia and birds, further evidence for the belief that those of the former do not contain nuclei; or, rather, that although their central may differ from their peripheral matter, the former does not stand in the relation of a nucleus to the latter, except in the blood-corpuscles of embryos. A chief point in the evidence is, that in the corpuscles of birds, small as they are, a nucleus may be demonstrated; and so plainly, that if such an one existed in the mammal's corpuscle, it could not escape notice when similarly searched for. Mr. Wharton Jones‖ also denies the existence of the nucleus in these corpuscles; and shows that the particles which are supposed to be nuclei exposed by the action of acetic acid on the corpuscles of mammalia, are only portions of albumen coagulated by the acid, such as may be produced by adding acid to liquor sanguinis or serum. At the same time it is not to be supposed that the corpuscle is homogeneous throughout; the central substance is evidently different from the peripheral, and Mr. Jones's opinion seems very probable, that the appearance of a nucleus depends on the walls of the corpuscle being thick, consisting of two layers,

* British and Foreign Medical Review, October, 1842, and Edinburgh Medical and Surgical Journal, October, 1843.

† The tendency of the pale corpuscles to keep with the liquor sanguinis is also shown by Mr. Gulliver, in his observations that fibrin obtained by washing, even when transparent and quite colourless, contains these corpuscles in great numbers. (Lond. and Edinb. Phil. Mag., Aug. 1842.) I have found that films of liquor sanguinis which has coagulated on healthy blood drawn under oil, are composed entirely of pale corpuscles held together like the cells of a tesselated epithelium.

‡ Proceedings of the Zoological Society, Dec., 1842.

§ London and Edinburgh Philosophical Magazine, August, 1842.

‖ *L. c.*

the outer transparent, colourless, and resisting; the inner softer and less resisting. Between the two, or perhaps in the inner layer, the colouring matter is contained. The existence of a true nucleus in the corpuscles of the lower vertebrata is not by this rendered doubtful; and Mr. Addison* has shown, in the use of *liquor potassæ*, a good method of demonstrating it in the corpuscles of the frog.

Many interesting observations have been made by both Mr. Wharton Jones and Dr. Carpenter, in support of the view that some of the corpuscles of the blood are a kind of "floating gland-cells."† The former believes that the office of the red corpuscles is to convert albumen into fibrin, elaborating it in their interior, and then, after the manner of gland-cells, dissolving and discharging their contents. The latter thinks it more probable that this office of converting the chemical compound into the organizable principle, is discharged by the pale corpuscles, and that the red corpuscles are the chief carriers of oxygen and carbonic acid. The arguments of each may be found in the last October and January numbers of this Journal. Whichever view be most true, it may be received as highly probable, that the corpuscles in the blood are the agents by which, with the aid of the oxygen imbibed in the lungs, the liquid portion is brought into a state fit for the nutrition of the tissues, and that this is their chief purpose.

Mr. Macleod‡ has described the development of the blood-corpuscles in the chick, dividing it into three stages. At first there are no particles in the blood except minute dark spherical granules. These gradually enlarge and become clear in their centres; but when they have arrived at the double of their original size, the central part of each becomes dull and then distinctly granular, while the border becomes defined, smooth, and clear. This completes the first stage, after which, in the second, the central granules disappear as if they had merged into one central clear nucleus, from which the external portion slowly separates, not at one side only, but all round. In this stage the corpuscle remains circular; but it becomes flatter, both on its surfaces and its edges, and a concave furrow forms between its outer border and the border of the nucleus. At the same time also it acquires colour apparently by the accumulation of colouring matter in the space between the nucleus and capsule. In the third stage, the corpuscle assumes the oval shape: first one side of both the cell and the nucleus gradually stretching out, and then the other, so that every part of the corpuscle becomes narrower except the middle. Coincidently with these changes, the furrow around the nucleus disappears, and the sharp edges of the borders are smoothly rounded off.

Mr. Macleod believes that all these changes are effected by a power dwelling in the granules, each of which developes itself into a cell. He has never seen any congregation of granules to form a cell, nor any multiplication of granules within the once-formed nucleus, nor any opening in the centre of the nucleus, nor any escape of corpuscles from it.§

### FIBRO-CELLULAR TISSUE.

Dr Todd and Mr. Bowman|| have thrown doubt on the received opinion concerning the structure of the fibro-cellular tissue. They regard it not as consisting of bundles of parallel filaments of definite size and structure, but as a substance which

* Transactions of the Provincial Medical and Surgical Association, vol. xi, p. 253.

† See Report, Oct. 1842, p. 12.

‡ London and Edinburgh Monthly Journal of Medical Science, Sept. 1842.

§ On the speedy and abundant development of microscopic vegetables (like those formed in fermenting fluids) in serum, or other albuminous fluids which have been neutralized by weak acids, diluted with water, and then exposed to the atmosphere, see MM. Andral and Gavarret's 'Recherches sur le Développement d'un Végétal microsc. dans les Liquides Albumineux,' read at the Acad. des Sciences, Jan. 30, 1843, in the Gazette Médicale, Févr. 11, 1843. There does not appear any ground for supposing that these vegetables (which are what Liebig supposed to be precipitated globules of albumen,) are, as some seem to think, a product of the blood; they are only formed under the same circumstances as other analogous vegetables are in other fluids exposed to the air.

|| Physiological Anatomy and Physiology of Man, p. 69.

has a tendency to split up almost *ad infinitum* in the longitudinal direction, and which has a filamentous appearance from streaks and creasings on its surface. [In small hard tendons, such as those of insects, this description may hold; they are compact and nearly homogeneous bands, with scarcely any appearance of a filamentous composition; but in the looser varieties of fibro-cellular tissue I cannot doubt the existence of bundles of distinct filaments.]

A few facts concerning the anatomy of tendons may be collected from the long discussions on tenotomy at the Parisian Academy of Medicine.*

### MUSCULAR TISSUE.

I have lately found a mode of attachmeut of the ultimate fibres of muscles to their tendons which has not yet, I think, been made known. It may be distinctly seen in the muscle torn out from the leg of a fly. Each of three tendons, which are planted in the proximal end of the last but one articulation of the leg, runs in a long straight and flat band up the interior of the next superior division of the limb, and receives on each of its edges the broad and somewhat rounded bases of the muscular fibres. These are arranged in a penniform manner, the base of each fibre on one side of the tendon corresponding to the halves of the bases of two adjacent fibres on the opposite side, like the leaflets of the pteris and some other ferns. The fibres are flat, and their extremities, instead of being ensheathed in the tendinous tissue, only adhere to the border of the tendon, and receive on their outer edges one or two finer tendinous filaments, as if for greater fixity.

Dr. Remak† has found that portions of the diaphragm, the heart, and the muscular walls of the larger vessels of many animals of all classes, will continue spontaneously contracting for as many as forty-eight hours after death. He has also pointed out their several modes of contraction, (?) which he distinguishes as creeping, undulating, peristaltic, and serpentine or zig-zag.

*Rigor Mortis.* An ingenious paper has been published by Ernst Bruecker‡ to prove that the rigor mortis is due to the coagulation of the fibrin which is effused from the blood-vessels in the liquor sanguinis for the nutrition of the tissues, (especially of the muscles,) but which at the time of death has not yet been assimilated. The paper is chiefly important for the numerous analogies which it points out between the coagulation and subsequent changes of the fibrin and the contraction of the muscles; analogies of which Mr. Hunter had already illustrated many, and some of the most important. But the explanation of the rigor mortis is rendered improbable by the observations of Mr. Bowman, which I can fully confirm, and which prove that the muscle is rigid because its fibres, or parts of them, are contracted, and contracted in the same manner as during life. And some examinations which I have made of the rigor mortis in the involuntary muscles, afford equally strong evidence of its being due in them also to the muscular contraction. I believe that *all involuntary muscles* pass into the continued and fixed contraction of the rigor mortis as soon as they cease to be irritable, and to contract under ordinary stimuli. This may be seen distinctly in many arteries, as well as in the digestive canal and urinary bladder; but the best examples are presented in the hearts of recently slain animals, or of men examined soon after apparent death.§ As soon as they cease to be irritable, the walls of all their cavities,

* Bulletin de l'Acad. Roy. de Médecine, Nov. 1842, Gazette Médicale, and other journals of the same date.

† Müller's Archiv, 1843, Heft ii. ‡ Ib. 1842, Heft iii.

§ Hearts in the state of rigor mortis are those commonly described as affected by concentric hypertrophy. Dr. George Budd proved some years ago that this appearance of an increased thickness of the walls with diminution of the cavities could not be due to disease of the heart; (Medico-Chirurg. Trans., vol. xxi, p. 296;) and I may add to the evidence which he adduced, (and which ought to have been taken as decisive of the question,) that in every instance the hearts of healthy oxen become affected with an extreme degree of *concentric hypertrophy* within an hour after they are slaughtered. I have little doubt also that the hearts of all persons pass into a similar state within a few hours after apparent death; certainly a large majority of the hearts examined within the first previously flaccid, gradually become firm and hard, draw in towards the base of the heart, and reduce or completely close the cavities. The heart thus rigid has almost exactly the form and other external characters of the heart when actively contracted during life. It is evident that this form, produced as it is by a drawing up towards certain fixed points of attachment of the muscular fibres, could not be acquired by the mere coagulation of either blood or liquor sanguinis within the tissue of the heart; and the only difficulty in believing that the rigidity of the heart and other muscles is due to a contraction comparable with that which occurs during more active life, must be from the seeming improbability that any tissue should maintain a vital contraction so long after apparent death as during the continuance of the rigor mortis. This difficulty, however, which would in any circumstances be more apparent than real, is almost removed by the facts already quoted from Remak.

CIRCULATION.

Dr. Marshall Hall,* in a paper on the 'Circulation in the Acardiac Fœtus,' has given proof that the pulsatory movement of the blood may, under certain circumstances, be communicated to the blood in a second set of capillaries. He placed the pectoral fin of an eel in the field of a microscope, and compressed it by the weight of a heavy probe. The movements of the blood in the capillaries became obviously pulsatory, their pulsations being synchronous with the contractions of the ventricle. He adduces this fact in support of the probability that the circulation in the acardiac fœtus is maintained by the force of the heart in the perfect twin fœtus, by which the blood is driven through the capillaries of the placenta into the umbilical vein of the acardiac fœtus, and thence through its venous capillaries into the aorta, and along the umbilical arteries to the placenta again. And the fact is equally important as an additional evidence of the general propagation of the force of the heart through one or even two sets of capillary vessels.

M. Poiseuille's observation of the influence of cold on the capillary circulation is mentioned in the last Report. (p. 44.) He has further shown† that the influence of some other agents is similar in organic and in inorganic capillary tubes. By adding successively acetate of ammonia, nitrate of potash, and alcohol to the blood, he found that the first two accelerated, and the last retarded its flow. They produced the same effects when added to serum which was made to pass through inorganic capillary tubes; as, indeed, might be expected, seeing that in both cases the bulk of the fluid moves, not upon the walls of the tube, but upon the layer of fluid which adheres and remains at rest upon the walls. Applying these results to determine the rate at which blood passes from one jugular vein to the other through the lungs, heart, &c., he found that the passage was made, in horses, in from eighteen to twenty-four seconds, when acetate of ammonia or nitrate of potash was added to the blood, but in from forty to forty-five seconds when alcohol was added.

According to Mr. T. Wharton Jones,‡ the congestion which succeeds to the temporary acceleration of the capillary circulation in an inflamed part, is due to the red blood-corpuscles adhering together (in the manner already described,) and to the walls of the vessels till stagnation occurs; and he has shown that the same arrest of the blood takes place when capillaries are touched with a solution of salt, or when a stream of carbonic acid is directed against those of the frog's lung. From these last facts he suggests with much probability that the stoppage of the circulation in the capillaries when certain salts are added to the blood, and that which takes place in asphyxia, depend on a similar adhesion of the corpuscles. With regard to asphyxia, his observations agree in their tendency with those of ten hours have more or less the appearance of being contracted, and many retain the appearance for twenty-four or thirty-six hours. In the majority relaxation and flaccidity return after the first day. But the rules as to time by which the rigor mortis is regulated in both the voluntary and the involuntary muscles have yet to be studied.

* Lond. and Edinb. Monthly Journ. of Med. Sc., June, 1843.

† Memoir presented to the Académie des Sciences, Janvier 9, 1843.

‡ Report, &c. Brit. and For. Med. Rev., Oct. 1842.

Dr. John Reid on the stagnation of the blood, independent of any apparent mechanical hindrance, when nitrogen is inhaled; and the action of carbonic acid in making the corpuscles cohere in rolls and assume the most favorable condition for the formation of a buffy coat, gives additional probability to the observations already quoted from Dr. Polli.

### RESPIRATION.

*Respiratory Movements.* MM. Beau and Maissiat,* have published some investigations in the physiology of respiration. Revising the forgotten opinions of Haller and Boerhaave, they have pointed out the very different characters of the respiratory movements in men, women, and children. They distinguish three types of these movements. 1. The *abdominal;* in which the visible movements are entirely in the abdominal walls, and especially in their anterior part, the ribs being unmoved, except when the body rests on the side. 2. The *inferior costal;* in which the movement takes place chiefly in the lower ribs, from the seventh inclusive downwards; those above the seventh moving very little, and the less, the higher they stand; and the lower end of the sternum ascending, though in a less degree than the ribs expand. 3. The *superior costal;* in which the movement is effected chiefly in the upper ribs, (especially the first,) which are carried upwards and outwards, and carry with them the clavicles and sternum.

In infants, and often to the third year of life, the respiration is of the abdominal type in both sexes. After the third year, the superior costal type is generally observed in girls, and the inferior costal in boys; and after puberty, the difference becomes more striking. Nearly all women breathe with the upper half of the chest, and nearly all men with the lower half and the abdomen. The mode of respiration in women has no connexion with their wearing of stays, but is probably adapted to the little capacity for breathing with the lower part of the chest during pregnancy. The difference is maintained, in general, even in dyspnœa; only, when it is extreme, a person whose natural respiration is according to any one of these types, may exhibit combinations of the movements proper to the others.

The quiet respiration of the rabbit and the cat is abdominal; their excited respiration is abdominal and inferior costal; that of the dog is always inferior costal; that of the horse is abdominal, except in sighing or when *blown,* when it becomes inferior costal, like that of man. These animals were used in experiments in which many of the actions of the respiratory muscles were observed.

On the anatomy of the osseous parts of the respiratory organs, the authors point out that the intercostal spaces are always proportionately widest between those ribs which are most moved in respiration; the superior are the wider in women, the inferior in men. In men, too, there is a remarkable distance between the sixth and seventh ribs, and the seventh and three following it often form a great projection. The articulations of the last two ribs with the spine are very lax, and their anterior ends being free, they follow the movements of the abdominal walls in which they are imbedded; they commonly descend in abdominal inspiration, and ascend in the inferior costal movement. The first rib is peculiarly moveable in women, and those who breathe like them; nearly, or quite immoveable in men and animals which breathe habitually with the lower ribs and abdomen. And herein is the solution of the question of the mobility or immobility of the first rib, as well as of that respecting the relative degrees of freedom of motion in the other ribs; they vary according to the peculiar type of the respiratory movements.

The shortness and early ossification and anchylosis of the first costal cartilage, make the sternum participate much more in the movements of the upper ribs than it does in those of the lower ones; hence, the antero-posterior enlargement of the chest in inspiration is much greater in women than in men. The increase of the intercostal spaces in inspiration is directly proportionate to their natural width; greatest, therefore, above in women, and below in men. In both, the increase is far greater anteriorly than it is posteriorly. In forcible expiration, the width of

* Archives Générales de Médecine, Décembre, 1842, Mai, Juillet, 1843.

the intercostal spaces may be reduced to considerably less than it is in ordinary expiration.

MM. Beau and Maissiat investigated also at great length the actions of the respiratory muscles, both by feeling and looking at them while in action, and by vivisections of dogs. Their conclusions, so far as the muscles are concerned in respiration, are briefly as follows, and many of them may be confirmed by observation on one's own person. *Intercostals:* In inspiration, they are elongated, and become hard and concave on their outer surface; in quiet expiration, they are moderately shortened, and become less hard and flat; in complex and forcible expiration, they become prominent and very short and hard. They are therefore muscles for forcible expiration, like their analogues, the oblique muscles of the abdomen; their hardness in inspiration is due to their being stretched; but their contraction (except by their elasticity) is only seen in forced expirations or in efforts.

[For many reasons, this conclusion must be considered very doubtful. The experiment on which the authors chiefly found their belief that the intercostals cannot raise the ribs, consisted in cutting through the pectoral muscles and the whole length of the intercostals between the sixth and seventh ribs on both sides: after this was done the lower ribs were still raised in inspiration (as they suppose) by the diaphragm. Perhaps no conclusion ought to be drawn from the results of such mutilation; but M. Debrou (Gazette Médicale, Jan. 3, 1843,) having repeated the experiment, with the addition of cutting the diaphragm from the ribs, and having found that the ribs were still raised in inspiration, maintains that the five lower ribs are thus raised by their intercostal muscles, and that the sixth, from which the intercostals above were cut away, is *pushed* up by the fifth. The following arguments appear to me conclusive in favour of the usually inspiratory action of the intercostals. 1. When the spinal cord is injured below the origins of the phrenic nerves and above those of the intercostal nerves, the ribs are very nearly motionless in respiration, for the intercostal muscles are paralysed though the diaphragm is active. 2. The upper ribs are chiefly moved in the superior costal respiration, though the diaphragm cannot act upon them. 3. The levatores costarum, which can act in inspiration alone, have an arrangement exactly analogous to that of the external intercostal muscles. 4. Whenever the intercostal muscles are affected by diseases in which the pain is increased by muscular contraction, there is an increase of pain in inspiration.] The authors believe also (and with more probability, for whatever be their ordinary action, the intercostals may in extraordinary circumstances, act in either direction,) that in forcible expiration they serve to make the whole walls of the chest rigid and resisting, so that they may not be distended by the eccentric impulse of the lungs, which are compressed on every side, and especially by the diaphragm. *Levatores costarum:* supposed (but improbably) to be not concerned in respiration, but to serve for maintaining the spine erect. *Infra costales:* probably muscles for forcible expiration, like the internal intercostals. (?) *Triangularis sterni:* a muscle of expiration, by drawing together the sternum and the costal cartilages. *Scaleni:* muscles of inspiration, especially in the superior costal type of movements, but chiefly flexors of the head. *Sterno-mastoid:* auxiliary to the scaleni in forcible inspiration. *Trapezius:* its upper border assists in forcible inspiration, its lower border in forcible expiration. *Levator anguli scapulæ:* acts with the upper part of the trapezius in violent inspiration. *Subclavius,* depressor of the clavicle after forcible inspiration. (?) *Latissimus dorsi:* its lower border acts in forcible expiration, as one may find by feeling the posterior wall of the axilla while coughing; at the same time it makes rigid those parts of the walls of the chest and abdomen on which it lies, and it presses in the lower ribs. *Serratus magnus:* acts in forcible inspiration, but chiefly (as was shown in a patient in whom it alone was paralysed,) it serves, by cooperating with the deltoid, in raising the arm. *Serratus posticus superior:* not a respiratory muscle, (?) but an extensor of the neck. *Serratus posticus inferior:* expiratory. *Pectoralis major:* its lower quarter is a muscle of inspiration, its upper three fourths form one of expiration, but it does not act except in dyspnœa. *Pectoralis minor:* its lower half acts habitually (?) as a muscle of inspiration.

As to the action of the diaphragm, the authors believe that it produces, 1. Elongation of the thoracic cavity, especially in the abdominal type of respiration. 2. Increase of the transverse diameter, by elevating and turning outwards the lower ribs, as in the experiment quoted in a preceding note; and this especially in the inferior costal respiration. 3. Occasionally, in infants, the depression of the costal cartilages. The second of these actions of the diaphgram is also described by M. Magendie.* The true mode of action is probably this: when the muscular fibres of the diaphragm contract, its central portion descends, and at the same time traction is exercised on the ribs at the peripheral ends of the fibres; and when the resistance to the descent of the diaphragm is greater than the resistance to an upward motion of the ribs, these are raised by the fibres which are attached to them, and whose direction, even in moderate inspiration, is nearly vertical. And this drawing upwards of the ribs is necessarily converted into a movement upwards and outwards by the limited and peculiar mobility of their attachments to the vertebræ and sternum. The third assigned action, that of the occasional depression of the inferior costal cartilages in children, is more reasonably ascribed by Mr. Alexander Shaw† to this, the most pliant part of the walls of the chest, being pressed in by the atmosphere when the other parts of the chest are expanded to a size which the lungs cannot attain, on account either of disease of their structure, or of obstruction to the free entrance of air through the larynx and trachea.

*Structure of the Lungs*. Mr. Addison‡ has given an account of the anatomy of the minute air-passages which, while it confirms nearly all that Reisseissen observed, is more complete, and very probably true. In the fœtus the ultimate bronchial subdivisions are tubular; they have a regularly branched arrangement, ramifying symmetrically in all directions, and terminating without anastomoses in closed extremities which are generally situated at the boundaries of the lobules. But when an animal has respired, the entrance of the air into the lungs distends the lobules, and the ultimate bronchial subdivisions undergo a great change. The membrane composing each of them offers only a feeble resistance to the pressure of the air, and is pushed forwards and distended laterally into rounded inflations, forming a series of communicating cells, which meeting on all sides those of the adjoining bronchial subdivisions, are moulded by the mutual pressure into various hexagonal and pentagonal forms. These distended passages (something like large beaded tubes) Mr. Addison calls *lobular passages;* and a section of them shows the *oval foramina* leading from cell to cell, which are so conspicuous in a thin layer of inflated and dried lung. The *air-cells,* according to this account, are the inflated parts of the *intralobular* bronchial subdivisions; and those of each lobule form a distinct system, having no communication with those of the adjacent lobules, except in the common trunk from which the intralobular bronchi of each system are derived. The air-cells are from 1-200 to 1-500 of an inch in diameter; and the oval foramina are from 1-60 to 1-150 of an inch or less in diameter. The blood-vessels lie upon each *lobular passage,* and between each two of them.

*Capacity of breathing*. M. Bourgery's examinations of the structure of the lungs are detailed in vol. XIV, p. 546. They may easily be reconciled with the more probable account of Mr. Addison, from which they chiefly differ in that the minutest branches of the bronchi are described in them as freely anastomosing, so as to form a series of labyrinthic canals; and that the constrictions of the tubes by which they are formed into cells or loculi are said to be due to annular vessels surrounding them. M. Bourgery§ has more recently examined the relations of the varying structure of the lungs in different ages and sexes to their functional capacity. The subjects examined were fifty males and twenty females, and the deductions are as

* Précis de Physiologie, p. 310.

† London Medical Gazette, Oct. 29, 1841.

‡ Philosophical Transactions, Part ii, 1842. An abstract, from which the above is taken, is in the Trans. of the Prov. Med. and Surg. Association, 1843, vol. xi, p. 281.

§ Paper read at the Académie des Sciences, Janvier 23, 1843; Arch. Gén. de Médecine, Mars, 1843; Gazette Médicale, and other French journals of the same date.

follows: 1. The measure of respiration (that is, I think, the proportion between the quantity of air which can be taken in by a forced inspiration, and the quantity which the lungs just previously contained) is always the greater the more youthful and lean the person is: strength and health do not in this regard compensate for youth. 2. The measure in males is twice as great as in females of the same age. 3. The function is at its highest point in both sexes at thirty years of age—the age which corresponds with the completest development of the aërial capillary plexus, or finest branches of the bronchi. At this age a forced inspiration increases the air in the chest from 2·5 to 4·3 litres in males, and from 1·1 to 2·2 in females. The boy of fifteen inspires two litres, the man of eighty, 1·35. 3. The volume of air necessary for an *ordinary* inspiration increases with advancing age; and this increase exactly represents the diminution of the energy of the pulmonary hematosis. 4. The capacity of the lungs for forcible inspiration increases from infancy to the age of thirty, doubling itself in twenty-three years. After thirty it diminishes one fifth in the first twenty years; one fifth more in the next ten; and nearly one half in the next twenty; and this gradual decrease of capacity for forcible inspiration is true of all persons, although one may have a greater general capacity of respiration than another of the same age. Hence the young person possesses a great capacity of respiration, as it were, in reserve; the old man has little, and is therefore unfit for great exertion.

*Exhalation of carbonic acid.* MM. Andral and Gavarret* state the following as the results of experiments made in sixty-two persons (thirty-six males and twenty-six females), to determine the quantity of carbonic acid exhaled in breathing: 1. At all ages beyond eight years the exhalation is greater in males than in females. 2. In males it regularly increases in quantity from eight to thirty years of age; from thirty to forty it is stationary or diminishes a little; from forty to fifty the diminution is greater; and from fifty to extreme age it goes on diminishing till it scarcely exceeds the quantity at ten years. 3. The quantity of carbon exhaled in the form of carbonic acid in one hour by males of different ages is as follows;—at eight years, 77·5 grains; at fifteen, 135 grains; at twenty, 176·7 grains; between thirty and forty, 189 grains; between forty and sixty 156 grains; between sixty and eighty, 142·5 grains; and in a man of 102 it was only 91·5 grains. 4. In females the same proportionate increase goes on to the time of puberty, when the quantity abruptly ceases to increase, and remains stationary so long as they continue to menstruate. When, however, menstruation has ceased, the exhalation of carbonic acid begins again to augment; and, then again, in advancing years, decreases as it does in men. Thus before puberty the quantity of carbon exhaled by girls in an hour is ninety-nine grains: and so it continues while the habit of menstruation continues; afterwards, from thirty-eight to forty-nine years of age, it increases to 130 grains; from fifty to sixty again falls to 113 grains; from sixty to eighty is reduced to 105 grains; and in a woman of eighty-two, was only ninety-three grains. 5. In amenorrhea the exhalation is always increased. 6. In pregnancy the exhalation is equal to that which is natural soon after the cessation of menstruation. 6. *Cæteris paribus,* the more robust a person is the more carbonic acid is exhaled; but the differences are not great. 7. The maximum of exhalation was in a strong man of twenty-six, who in an hour exhaled carbonic acid containing 218·5 grains of carbon; the proportionate minimum in a weak man of forty-five, who exhaled in the same time only 139·5 grains. 8. The influences of the weights of persons, of the capacities of their chests, and of the extent of the respiratory movements, are not great.

DIGESTION.

*Structure of the teeth.* Mr. Lintott† has pointed out the fact of a regular and

* Recherches sur la quantité d'Acide Carbonique exhalé par le Poumon.—Paris, 1843.

† On the Structure of the Human Teeth.—Lond. 1843; abstract in the Lancet, June 24, 1843. There are some excellent illustrations of the mode of growth of the teeth, in Mr. A. Shaw's paper "On the effects of rickets upon the growth of the skull," in the Medico-Chirurg. Trans. 1843, vol. xxvi.

constant formation of a layer of bone or, probably, of imperfect ivory like what Mr. Nasmyth has called ossified pulp, within the pulp-cavity of the human tooth, after the age of twenty years, independently of any wearing down of the enamel. The layer is thickest at the orifice of the dental cavity, and gradually diminishes as it descends into it till it is lost upon the walls; its thickness increases with advancing age. He remarks also that the part which is by far the most frequent seat of the commencement of decay in the molar teeth is the groove which separates the tubercles of their crowns, and at which the operculæ (according to Mr. Goodsir) meet when the papillary is changed into the capsular stage of development. These grooves are first affected as regularly in the upper as in the lower jaw; as if they were from the first imperfectly developed: [that is, probably, they are liable to the imperfections of parts last formed, such as are often seen in the other *lines of median* or *central fusion.*]

*Salivary secretion.* Dr. Budge* has found that after extirpation of the parotid, submaxillary, and sublingual glands in a dog and a rabbit, the secretion of saliva continued; its characters remained the same, and no function was disturbed. [The experiments add probability to the opinion that the labial, buccal, palatine, and other glands which the experimenter left behind, are salivary glands.]

A case of a kind of metastasis of the salivary secretion is related by Dr. Roelants,† and is interesting in its relation to the general physiology of secretion. A man, eighty-two years old, had an attack of bronchitis, with fever, followed by suppuration around and probably in one of the parotid glands. The abscess was opened, and two months after a large mass of chalk-like substance was discharged. The abscess soon healed, and he recovered his health; but now, whenever he masticates, saliva flows freely from the skin of the cheek and temple of the side formerly diseased. As soon as he begins to eat, the skin becomes very full of blood, and hot; and gradually drop after drop of clear fluid, with all the characters of saliva, collects on its surface, and runs down the cheek and neck, and continues to do so just as long as he continues eating. His health is not disturbed, and the saliva-secreting surface of the skin is natural in its texture.

*Anatomy of the pharynx.* Professor Mayer of Bonn described some time ago‡ a *bursa pharyngea* in many mammalia. He has since found it several times in men. It lies in a corresponding position to that which it occupies in the mammalia, namely, in the middle line in the mucous membrane covering the body of the sphenoid bone, just behind the posterior border of the vomer. It is sometimes large enough to hold a cherry-stone, and in one case was double. He thinks it probable that in other mammalia, in which the bursa is larger, it may sometimes communicate with the sphenoidal sinuses.§

*Functions of the stomach.* MM. Sandras and Bouchardat,|| assuming that, in general, dissolved substances are absorbed by the veins of the stomach, while those that are insoluble are taken into the lacteals, believe that they have proved that the chief classes of aliments are thus disposed of: 1. Fibrin, albumen, caseum, gluten, and the gelatinous tissues are dissolved by the aid of hydrochloric acid; [and, probably, of pepsin.] A mixture of six parts of this acid with 10,000 of water they found sufficient to make all these principles swell up into translucent

* Schmidt's Jahrbucher, Bd. xxxv, Heft 3. Dr. Budge's conclusions on the chemical and other characters of the saliva are confirmatory, so far as they go, of the statements in the essays by Dr. Wright, (Lancet, March 5, 1842, and following numbers;) of which I must regret that those relating to the composition of the saliva were published before the date at which this report commences. Many of them are confirmed also by Lehmann. (Schmidt's Jahrbucher, 1843, No. viii, p. 156.) He however states that he has always found sugar altered by saliva, lactic acid being produced by their contact at 95 deg. Fahr. See also on this subject the review of Schultz, Ueber die Verjungung, &c. in vol. XVI, p. 232.

† Heije's Archief voor Geneeskunde, 1842, St. iv.

‡ Froriep's Notizen, April, 1840.

§ Neue Unters. aus dem Gebiete der Anatomie und Physiologie—Bonn, 1842.

|| L'Expérience, Février 3, 1843, from a paper read before the Académie des Sciences.

masses, and *sometimes* to dissolve them. 2. The starchy and saccharine principles are converted wholly or in part into lactic acid, and in that form are absorbed in the stomach. 3. The fatty matters are insoluble, and pass into the intestines, where they are taken up by the lacteals, and form the greater part of the chyle. The experiments which were performed to confirm these opinions before the reporters to the Institute did not succeed well; but if they had done so it would still be hard to explain how the albumen and fibrin can be formed in the chyle from fatty matter alone. Still that some of the starch of food may be transformed and absorbed in the stomach is confirmed by the experiments of Dr. Percy.* These make it probable, 1, that sugar is formed in the stomach by the digestion of starch or wheat flour, though neither these experiments, nor any others yet performed can afford demonstrative evidence of it; 2, that the dextrin into which the starch is first transformed may be at once absorbed, so as to reduce the quantity of sugar which is formed; and 3, that the sugar which is formed must be quickly further changed or absorbed. The latter is the more probable conclusion, and best accounts for the very small quantity of sugar which is ever found after feeding on starch. Lastly, Dr. Percy suggests, that in the cases in which Dr. McGregor found sugar in the stomachs of those diabetic patients who for several days had had only animal food, it might be formed by the oxydation of the fat which is constantly being absorbed from the body during emaciation.

*Composition of the bile.* Dr. Kemp,† by careful elementary analysis of the bile of the ox, has corroborated Demarçay's opinion that it is essentially a true chemical compound of an electro-negative body with soda. But he holds that this body is neither the *choleic acid* of Demarçay, since it is not precipitated from the soda by acetic acid, nor the *bilin* of Berzelius, because it is not precipitated from the soda by carbonic acid. He has therefore given it the name of *bilic acid*. It has a peculiar bitter-sweet taste, and in mass resembles a fine resin. It is soluble in every proportion in water. In a subsequent paper‡ he has shown that a much greater difference than is usually imagined is effected in the bile while in the gall-bladder. Bile from the hepatic ducts of an ox was destitute of the bitter taste of cystic bile; its smell also was different. It chiefly consisted of two different electro-negative bodies, separable by alcohol, and each combined with soda.

### ABSORPTION.

M. Lacauchie§ describes the intestinal villi as possessing during life a power of alternately retracting and elongating themselves by a kind of vermicular motion, which he believes to be influential in the propulsion of chyle. And his account, so far as these movements are concerned, is confirmed by MM. Gruby and Delafond,|| who have observed them in the recently-slain horse, dog, and rabbit. They add that besides the movements of retraction and elongation, the villi are capable of moving laterally in all directions, and that their epithelium-cells bear ciliæ.

Some experiments by Dr. Behr¶ may serve, perhaps, to explain somewhat of that which was supposed to depend on an elective power of absorption possessed by the lymphatics, and certainly have added much to the probability that the force by which the lymph is carried along the lymphatics is that of the contraction of their walls.** It has been long known that the lymphatics will not convey certain

* "Case of Diabetes," Medical Gazette, April 7, and following numbers, 1843.

† London Medical Gazette, Dec. 16, 1842, and March 3, 1843.

‡ Medical Gazette, May 5, 1843.

§ Paper read at the Académie des Sciences, May 15, 1843, see Comptes Rendus and contemporary journals.

|| Paper read and reported as above, June 5, 1843. MM. Gruby and Delafond assign to the epithelium-cells of the villi nearly the same offices as are, with much more probability, assigned by Mr. Goodsir to the transitory cells developed within the villi.

¶ Henle and Pfeufer's Zeitschrift, für ration. Medicin. Heft i, 1842, and Schmidt's Jahrbucher, Heft iii, 1843.

** See last Report.

substances, especially narcotic poisons, while they do carry others. If, for example, the animal's abdominal aorta be tied so as to stop the circulation in its posterior extremities, and ferrocyanate of potass be inserted in a wound in one of them, it is absorbed and carried into the blood by the lymphatics, and is found again in the urine. But if, under the same circumstances, a narcotic poison is put in the wound the animal is not killed by it; and it was supposed that the lymphatics in this exercised some kind of choice. The results of Dr. Behr's experiments are these: 1. Acetate of strychnine was introduced into a wound in an animal's leg, while the circulation was uninterrupted, and death, with convulsions, &c., occurred in five minutes. 2. Ferrocyanate of potass was introduced into a similar wound, and ten minutes after acetate of strychnine into another wound: in four minutes the animal died of the poison, and the ferrocyanate was found in the urine. 3. The same substances were introduced together into a wound in the leg: the animal died poisoned, and even sooner than before, and the salt was found in the urine. 4. The abdominal aorta was tied below the renal arteries, and when the hind limbs were paralysed the acetate of strychnine was put into a wound in one leg and the ferrocyanate of potass into a wound in the other. After two hours and half there were no signs of poisoning but on killing the animal the salt was found in the urine. 5. The abdominal aorta was tied as in No. 4, and the acetate of strychnine and ferrocyanate of potass were introduced into the same wound. The animal showed no signs of poisoning, and the salt could not be found in the urine. This last experiment was several times repeated, and, with unimportant variations, with a constantly similar result. It would follow, therefore, that when the circulation in the blood-vessels is stopped, the lymphatics can absorb and convey to the blood ferrocyanate of potass, but not acetate of strychnine; and that when the two substances are applied to them together it can absorb or carry neither. Hence it is supposed that the force by which the lymphatics convey fluids is that of the contraction of their walls, and that they are paralysed by the direct contact of narcotics, as other involuntary muscles are.

Mr. George Robinson* has related some experiments in evidence that the absorption of blood-vessels depends on a force generated by and proportioned to the velocity of the blood which is moving in them. He compares it to that force with which water or any other fluid traversing a main tube will draw fluid through a side branch, even against the weight of a considerable column. He has often repeated this well-known experiment, and has added proof that the same force will act in the same way through one or more membranes. Having filled a wine-glass with coloured fluid, and having connected its contents, (by means of a bent tube twelve inches long and $\frac{1}{14}$ of an inch in diameter, and having one of its ends covered with membrane,) with the interior of a pipe half an inch in diameter, he found that within five minutes after the stream had begun to flow rapidly through the last-mentioned pipe, the whole of the air present in the smaller tube was absorbed, and its place supplied by the coloured fluid, which had risen from the glass. In another experiment the fluid from the glass was raised through a shorter tube to the membrane, and was made to flow in a slow but constant stream towards the fluid, passing through the larger pipe.

### TRANSFORMATIONS OF NUTRITIOUS SUBSTANCES.

Among the numerous papers written on the transformations which the food undergoes in its passage in the body, the most interesting and almost the only ones which afford any definite conclusion are those relating to the formation of fatty matters from the saccharine and starchy principles. It seemed to be proved by Huber's experiments on bees that wax could be formed by them out of pure sugar or honey; for when their food contained nothing but one of these they formed their combs as usual. M. Dumas, who had opposed Liebig's deductions from these facts, suspected that the wax might be formed from the fat which the bees had in their own bodies before they commenced their purely saccharine diet. He therefore,

* Lancet, May 27, 1843.

with M. Milne Edwards,* repeated the experiment, and in a successful trial obtained the following results: 1988 bees were inclosed in a hive, and from an analysis of the bodies of 117 from the same stock it was estimated that the bodies of the 1988 contained 3·218 grammes of fatty matter. The honey on which they fed contained $\frac{8}{10000}$ of waxy matter. The experiment was continued thirty-one days, and the bees consumed 834·889 gr. of honey, and produced 11·515 gr. of wax, or at the rate of 0·0064 for each bee. After the experiment 105 bees were analysed, and yielded 0·442 gr. of fatty matter, or at the rate of 0·0042 gr. each. Thus the fatty matter preexisting in each bee was 0·0018 gr., and the quantity furnished to each in its food was 0·0038: but each produced in thirty-one days 0·0064 gr., and each at the end contained 0·0042, giving a total of fatty matter 0·0106, and an excess, which must have been formed by transformation of the food, equal to 0·00742 gr. per bee.

The old view of the production of the oleaginous constituents of the bodies of herbivora by the transformation of the saccharine and amylaceous principles of their food is thus confirmed; and the evidence is the better for its being honestly published by one who had been the chief opponent of the view. Connected with it is a fact recently observed by MM Pelouze and Geliş,† that under certain circumstances, butyric acid is formed during the fermentation of sugar. By the action of the acid thus obtained upon glycerin they formed also butyrin, another of the constituents of butter. Still, however, the results of the experiments on which Dumas' former opinion was founded are important, as proving that the several articles of food of the herbivora contain a much larger proportion of fatty matter than had been imagined. In maize and other grains, for example, he has found from 7 to 9 per cent., and in grass, hay, &c. considerable proportions, which in all probability contribute to the formation of the fat, though they are not its only source.

*Assimilation.* In the last Report‡ some remarkable observations were referred to, proving the analogies between the forms assumed by certain inorganic precipitates such as those of the carbonates of lime and iron, and the forms of the nuclei and cells of organic tissues. Now, Dr. Hermann Jordan,§ of Saarbrück, has called attention to the phenomena of the reparation of damaged crystals, as bearing analogy to the repair of injured organized bodies. The facts which he establishes are these: 1. Any portion of crystal—whatever surfaces, angles, or edges may have been removed from it—may, under proper circumstances, repair itself into a complete individual; that is, restore itself to the same form which it would have had if no injury had been done to it. 2. At the same time with the reproduction of the truncated part, a growth of the whole crystal takes place: *but the effort of the formative act is especially directed to replace the lost part.* 3. The effort at reparation stands in a direct relation to the extent of the loss, and decreases in proportion as the loss is replaced. 4. He points out the mode in which the process of reparation takes place, and concludes that his examinations "have demonstrated the tendency of individuals to maintain their integrity and to replace material losses by the formation of more matter, according to the type of their original form, as a phenomenon to be observed as well in inorganic as in organic nature —as a phenomenon which belongs to the individual as such, whether it have a membered body or the simple structure of a crystal."

ORGANS OF ANIMAL LIFE.

*Chemical composition of bone.* The following are the results of the most recent and careful analyses of human bone, by Marchand and Lehmann. In both cases

* Paper read at the Académie des Sciences, Paris, Sept. 18, in the Comptes Rendus, and contemporary journals. Before the publication of these experiments many controversial papers of much interest had appeared, many of which may be found in the Annales de Chimie et de Physique, and the Annalen der Chimie und Pharmacie, January, April, &c. 1843, as well as in the contemporary numbers of the Gazette Médicale, the Medical Times, the Annals of Chymistry, &c.

† Paper read and reported as above, Juin 12, 1843.

‡ See p. 12. § Müller's Archiv, Heft i, 1842.

the bones were deprived of fat and periosteum : in each the average of six examinations is given; Marchand's* were made on thigh-bones, Lehmann's† on the long bones of the arm and leg.

| | | | Marchand. | Lehmann. |
|---|---|---|---|---|
| Organic matter | Cartilage insoluble in H.CL. | 27·23 | 33·26 | 32·56 |
| | ,, soluble ,, | 5·02 | | |
| | Vessels | 1·01 | | |
| Phosphate of lime | | | 52·26 | 54·61 |
| Fluate of lime‡ | | | 1· | |
| Carbonate of lime | | | 10·21 | 9·41 |
| Phosphate of magnesia | | | 1·05 | 1·07 |
| Soda | | | 0·92 | 1·11 |
| Hydrochlorate of Soda | | | 0·25 | 0·38 |
| Oxydes of iron and manganese and loss | | | 1·05 | ·86 |
| | | | 100· | 100· |

The following are average relative proportions of organic and earthy matter, collected by Lehmann from his own and the analyses of two other observers.

| | Sebastian. | Lehmann. | Frerichs Compact bone. | Frerichs Spongy bone. |
|---|---|---|---|---|
| Organic | 36·66 | 32·28 | 31·2 | 37·82 |
| Earthy | 63·34 | 67·72 | 68·8 | 62·18 |

All found that the earthy matter increases with age.

In a memoir on ancient and fossil bones, M. Gerardin§ states that the degrees of alteration which buried bones undergo depend almost entirely on the degrees in which the soils are exposed to air and moisture. They always lose more or less of their animal matter; and sometimes, when they lie in a soil traversed by streams of water, it is completely removed: the ammonia proceeding from the part first decomposed saponifies the rest and makes it soluble. In human bones long buried and in fossil bones there is always more subphosphate of lime than in recent bones; in *human bones* long buried the proportion of carbonate of lime is generally diminished, in fossil bones it is increased. In fossil bones also there is always some fluate of lime; in human bones, under whatever circumstances, there is none: it seems to be introduced into fossil bones by infiltration from without, and its presence may be depended on as a sign that a bone is really fossilized.

*Structure of bone.* Dr. Fleischmann‖ has described the minute structure of *vegetable ivory*, from the fruit of the *manicaria saccifera*, (Gärtner,) a species of palm growing near the coast of Guiana, as being closely analogous to that of bone, at least in regard to the *corpuscles* which it presents. It possesses also, he says, somewhat of the chemical properties of bone. Thin sections exhibit the most beautiful structures, like the bone-corpuscles, except that they are more regular, and lie within regularly-formed cells, of which they appear to be the nuclei. Branches like the calcigerous canals proceed from each corpuscle, but do not give off smaller branches nor extend beyond the wall of the cell; each branch ends within the cell-wall in a bluntly-closed extremity. He believes that there is the same arrangement in true bone; that each corpuscle has, like a nucleus, a distinct cell-wall around it, such as he has figured in a section of bone from a child; that the canals of the corpuscles are unbranched, and that they end within the cell-walls, having only an appearance of anastomosis with the canals in adjacent cells.¶

* Journal der Prakt. Chemie, Bd. xxvii, p. 83.

† Schmidt's Jahrbucher, 1843, No. vi; see also the Chemist, 1843, Nos. 1, 2, and 3.

‡ See the next paragraph from M. Gerardin, whose analyses confirm those of Mr. G. O. Rees, in denying the presence of fluate of lime in human or any but fossilized bones.

§ Report from the Académie des Sciences, Gazette Médicale, Oct. 15, 1842.

‖ Müller's Archiv, 1843, Heft iii, p. 202.

¶ In these two last opinions he is certainly wrong; the first is in accordance with the

The chapter on 'Bone,' in the 'Physiological Anatomy' of Todd and Bowman, contains by far the best plates yet published of the minute osseous structure. The ultimate structure also is there described, from preparations made by Mr. Tomes, to be granular. The ultimate granules vary in size from 1-6000 to 1-14000 of an inch; they are oval or oblong, and cohere firmly, possibly by the medium of some second substance. In some instances Mr. Tomes has met with a very minute network, which seems adapted to receive them in its interstices; but this, he considers, requires confirmation.

*Process of ossification.* In the same work is a description of the process of ossification, which is, in several points, new and interesting, (p. 117.) In the vicinity of the point of ossification the nucleated cartilage-cells (which usually are scattered irregularly) arrange themselves in linear series, which run down, as it were, to the ossifying surface. At first the series are small and not regular, but nearer to the ossifying part they form rows of twenty or thirty. The cells in these rows are closely compressed, and their nuclei seem flattened. The lowest rows dip into and rest in deep narrow cups of bone, formed by the osseous transformation of the intercellular substance between the rows, and as ossification advances these cups are converted into closed areolæ or *cancelli,* with extremely thin lamelliform walls. Immediately upon the ossifying surface nuclei, which before were closely compacted, separate considerably from one another by the increase of material within the cells: they also often enlarge and become more transparent. Deeper in the new bone the lamellæ which inclose the cancelli, and which were formed by the ossification of the intercellular substance, are found thicker and more like perfect bone: they include in their substance elongated oval spaces, which, except that they are roughly granular, exactly resemble the ordinary *bone-corpuscles,* and which are evidently the nuclei of the cells of the temporary cartilages. The curvilinear outline of the now ossified cells of these nuclei can often be discerned. Within the cancelli only a few cells can be detected, these cavities (of the cancelli) being chiefly occupied by a quantity of new substance, consisting of granules, and resembling a formative blastema or basis. It thus appears that after the ossification of the intercellular substance, (by which are formed the lamellæ which are the walls of the cancelli,) the rows of cartilage all arrange themselves on the inner surface of these newly-formed cancelli, and are ossified, with the exception of their nuclei, which remain granular, and subsequently form the corpuscles of bone; and that a new substance or blastema appears within the cancelli, from which, probably, vessels are developed, and the further steps in the growth of the bone proceed.

### NERVOUS SYSTEM.

*Minute structure.* Remak* says that on the axes of each of the larger primitive tubes of the abdominal nervous cord of the River Cray Fish (*astacus fluviatilis*), there is, in the recent state, a winding bundle of extremely delicate fibres, occupying one third or one fourth of the whole diameter of the tube. The fibres of this *central fasciculus* are smooth, parallel, without branches or anastomoses, and less than 1-5000 of an inch thick. They may be seen distinctly when the tubuli are injured: some of them often protrude from the broken extremity. They are found, however, only in tubules from 1-60 to 1-30 of a line in diameter; smaller tubules than these either appear translucent or contain a fine granular substance, and none but the smaller tubules, such as these, are found in the nerves and nervous trunks near the abdominal cord. The spaces between the central fasciculi and the walls of the larger tubules are filled by a clear, colourless fluid. The relation of the central fasciculus in the large tubules of the nervouscord to the central substance of ordinary nerves (the *primitive band* of Remak,) is uncertain.

*Repair and union of nerves.* Dr. Bidder,† of Dorpat, has made several experiments to determine whether nervous filaments of originally different functions can be made to unite. He experimented on the lingual and hypoglossal nerves manner in which Henle supposes the corpuscles and their canals to be developed by a deposition within cells, having in each a central cavity (the corpuscle) and interstitial passages or pores (the canals). See his Allgemeine Anatomie, p. 182.

* Müller's Archiv, 1843, Heft iii, p. 197. † Ib. 1842, Heft i and ii.

of dogs, but the results were inconclusive. They tended, however, to prove that such a union does not take place; for in several of the cases the connected portion of the two trunks were found, on subsequent examination, disparted, and each had united again with that portion of itself from which it had been separated. It was found that a sufficient union for the restoration of function can take place in three or four months, although a portion of a nervous trunk eight lines in length has been completely removed.

*Reflex action.* Some evidence in favour of the view that the nerves of the excito-motory system form a system distinct from those conveying sensation and volition, is afforded by the investigations of Mr. Newport.* He finds that in the myriapoda the fibres which correspond to the true spinal cord in vertebrata are distinct from those connected with the cephalic ganglia. They form part of the cord in the intervals between the abdominal ganglia, and may be traced from the periphery into the several ganglionic centres, from which they pass backwards along the cord until they arrive at the next ganglion, from which they pass again to the surface of the body. Now there is reason to believe that the ganglia are not sensitive; for the reflex acts, which are repeatedly performed after the removal of the head or destruction of the central ganglia, are performed without any appearance of volition being exercised in them and always in one and the same manner.

*Influence of the nervous centres.* Professor Volkmann,† one of the most accurate experimenters of modern times, has occupied himself in testing the value of those experiments which are supposed to prove the direct influence of the central nervous organs upon the movement of the viscera.

With regard to the part of the centres on which the movements of the heart depend,—Volkmann shows that in fresh-slain animals the movements of the heart are so completely irregular, even when left to themselves—in one half minute hurried, in the next retarded, then stopping for one or more minutes and then of themselves going on again—that it is impossible to determine the influence of any supposed excitant of the brain or spinal cord. From a great number of experiments, very carefully conducted, no fixed result could be arrived at except this, that the existence of any direct influence exerted on the heart by irritating the nervous centres is as yet altogether doubtful.

He has come to the same conclusion, upon equally good negative evidence, in regard to the effects supposed to be produced on the motions of the stomach and intestines by irritating the brain and cord. He could find no such influence exerted. The motions of the alimentary canal often entirely cease for a long time, and are then of themselves renewed; but when once they have entirely ceased, no irritation of the nervous centres can reproduce them, although it is certain that after the canal ceases to move the nervous centres are still irritable. He peremptorily denies Budge's experiment in which he believed that though the peritoneum of the abdominal walls was left, the intestines moved when the central organs were irritated and ridicules the *active inflation* of the stomach, which Budge supposed to be thus produced. With equal positiveness he denies the truth of Budge's statements respecting the elevation and expansion of the testicle when a part of the cerebellum is irritated. In repeated trials he could produce no such effects as Budge reports, in either the digestive canal or the testes.‡ His conclusion is, "I am far from

* On the structure, relation, and development of the nervous and circulatory systems ............... in the Myriopoda, &c., Transactions of the Royal Society, 1843. See also Br. and For. Med. Review, vol. XVI, p. 160, et seq., in which, in the review of Arnold and M. Hall on the reflex theory, both this and all the other evidence for the *anatomical distinctness* of the excito-motory system are adduced, with the exception of the observations by Van Deen presently to be mentioned. [Since the preceding was in type I have been favoured with the perusal of the further analysis of Mr. Newport's works contained in an earlier part of this Number, to which I must refer as affording a more complete account of them than could be inserted in the text.]

† Müller's Archiv, 1842, Heft v.

‡ The accuracy of several others of Dr. Budge's experiments and deductions is impugned by Dr. Stilling in Haeser's Archiv, 1842, Heft i, and in Schmidt's Jahrbucher, 1843, Heft ii, iii, &c.

denying that the central organs exercise an influence on the motions of the viscera, for pathological observations make that certain. But they do not prove that this influence is a direct one ; and experiments on living or fresh-slain animals prove it still less."

*Brain.* In a very valuable contribution to the statistics regarding the weights of organs—of which, however, the greater part can as yet serve only to add to the necessary heap of evidence—Dr. John Reid* has made it probable; 1. That the cerebellum does not attain its maximum weight at seven or a few more years of age, though it does attain it sooner than the other organs, and the size of the whole brain, in proportion to the entire body, is greater in the child than in the adult. 2 That the average weight of the cerebellum compared with that of the whole brain, is a little greater in the female than in the male. 3. That though the male brain is on the average heavier than the female, yet in proportion to the weight of the whole body, it is rather less heavy. 4. That the brain does not in emaciation diminish in the same proportion as the rest of the body does.

M. Parchappe† has shown from his measurements and weighings that in regard to *the size of the head:* 1, that of males is to that of females as 16,128 : 15,294 ; 2, it increases gradually to the sixtieth year, chiefly through enlargement of the frontal sinuses, and after that time diminishes; 3, it is in same measure proportioned to the stature. And in regard to the *size of the brain :* 1, that the male is to the female brain, on an average, as 156 : 125, and that in weight they have about the same proportion ; 2, that it increases to the fortieth year, and then decreases to the seventieth ; 3, that it bears some proportion to the stature ; 4, that the intellect is not absolutely proportioned to the size of the brain, but is proportioned to the size of the hemispheres, and especially to the extent of their surfaces.

Dr. George Burrows‡ having repeated the experiments of Dr. Kellie and performed others has shown, in opposition to the opinions commonly entertained, 1st, that the brains of animals bled to death are deprived of their blood, and rendered pale and anæmic ; 2, that the quantity of blood in the head is greatly affected by posture and gravitation ; 3, that in death by apnœa there is intense congestion of the cerebral vessels. And from these facts and from several considerations he deduces that the opinion that the quantity of blood within the cranium is at all times the same is untrue. Admitting that the total contents of the cranium must be at all times nearly the same, the cerebro-spinal fluid is rapidly removable from one site to another, and capable of being altogether removed by absorption ; so that this fluid may be regarded as supplemental to the other contents of the cranium—at one time giving place to the increased quantity of blood, at another making up for the deficiency of blood in the vessels and in the same manner varying according to the actual quantity of nervous substance.

*Spinal cord.* From Dr. Knox§ we have a description of the spinal arachnoid, maintaining that the account usually given of it and of the absence of any regular communication between its cavity and that of the cerebral ventricles is correct, in opposition to the descriptions of Dr. Sharpey and Mr. Ellis.

Drs. Stilling and Wallach‖ deny the existence of globules in the gray matter of the spinal cord, and say that those which have been held to be globules are fragments of divided nerve-tubes. These tubes of the gray matter they describe as differing from those of the white in being of less diameter, having thinner external walls, and being differently coloured. The course of some of them is longitudinal ; that of others transverse, and these are continued into the white substance of the cord, crossing the direction of its fibres, but never uniting with them. The

* London and Edinb. Monthly Journal of Med. Science, April, 1843.

† Gazette Médicale, Octobre 8, 1842.

‡ Medical Gazette, April 28, and May 5, 1843.

§ Medical Gazette, June 23, 1843.

‖ This account is taken from an analysis of their work (Untersuchungen über die Textur des Rückenmarks, Leipzic, 1842, 4to,) in the Allgem. Medic. Central-Zeitung, Febr. 22, 1843. I have not had time to see more, since obtaining the original, than that the analysis given of it is generally correct.

filaments of the roots of the nerves are continuous, not with the white filaments or tubules of the cord, but with these its transverse gray filaments.

In some "Additional experiments on the spinal marrow," Dr. Van Deen* mentions two which he has frequently repeated in the presence of competent judges, to prove that the nervous fibrils of the limbs of the frog do not proceed to the brain, but terminate in the spinal marrow. In the first experiment the whole spinal marrow of a frog is exposed, and *all* the roots of all the nerves which go to the fore-legs and abdomen are cut on both sides; the marrow is then divided a little above the place where the nerves of the fore-legs are cut through, and its divided end being gently raised, a portion of glass or paper is pushed under it; but this is done only for the convenience of the further cutting. If now small portions of the spinal marrow are cut off successively from above downwards with great care and without shaking, no muscular movements are excited in the hind-legs; and the sections may be continued to within a little of the place at which the first lumbar nerve leaves the spinal marrow. It is on cutting this part that one first sees muscular motions in the upper part of the thigh; and as one goes on cutting lower down they ensue in both the hind feet.

In the second experiment all the roots of all the nerves of the hind legs are first cut on both sides of the cord, and a portion of paper being put under the lower end of the cord, pieces of the cord are cut off in succession from below upwards. No signs of pain are induced, nor (even when the animal is beheaded) is any motion of the fore-legs excited, till one comes to that part of the cord from which the undivided roots are given off.

These two experiments prove, says the author, 1st, that the primitive fibrils do *not* pass through the spinal cord to the brain, since if that were the case every division of the cord in the first experiment must produce motion in the hind-legs, and every division in the second must have excited pain or, at least, some motion of the fore-legs; and 2dly, that the spinal marrow is not capable of propagating any irritation communicated to it to a great distance through itself, unless nerves are connected with it.

An interesting case bearing upon the physiology of the several columns of the cord is related by Dr. Webster.† There was complete loss of voluntary motion in the trunk and limbs, with retention of natural sensibility in them, and active reflex movements, in consequence of softening of the whole thickness of the middle part of the cervical portion of the spinal cord. The case is inexplicable in the present state of knowledge, unless we believe that Van Deen's experiments are conclusive which seemed to prove that the gray matter of the cord can, generally speaking, convey centripetal impressions to the brain, but not centrifugal impressions from it, and, besides, suppose that in this case the morbid change of the gray matter was not so complete as wholly to interrupt its functions. There are other cases sufficient to prove that considerable degrees of softening and other changes of structure of the cord may exist without complete loss of function.

Mr. W. F. Barlow‡ has published some good remarks on the influence of the impressions from sudden changes of temperature in producing reflex movements.

*Particular nerves. Optic.* Professor Erdl§ considers that he has traced the fibres of the optic nerves through the following long course: From the optic tract they diverge and expand in the substance of the thalami, then again converge towards the anterior part of the thalami and unite into a cord distinguished by its white colour, which descends into the corpora albicantia, forms a loop in them, and then turning upwards and forwards ascends through the anterior crura into the body of the fornix. From the fornix the fibres are continued into its posterior crura,

* Tijdschrift voor Natuurliche Geschiedeniss en Physiologie, 1842, vol. ix.

† "Case of paralysis," &c. in the Medico-Chirurg. Trans. 1843, vol. xxvi, p. 1; also Report in the Lancet, Nov. 1843.

‡ Lancet, May 13, 1843.

§ Neue Medic. Zeitung, 1843, No. vii, and Oesterreichische Medic. Wochenschrift, May 25, 1843.

and into the corpora fimbriata, in which they descend into the pes hippocampi on each side, whence again they ascend in the tapetum to the posterior part of the corpus callosum, in which the fibres of the two sides again unite. He describes also the generally admitted fibres passing from one retina to the other through the anterior part of the optic commissure; and he supposes that the peripheral ends of these fibres are connected with those of the fibres whose course is described above, so as to form a kind of closed system or nervous ring.

*Third nerve.* Dr. Fäsebeck,* of Brunswick, describes a branch of the superior division of the third nerve, which is given off soon after that nerve enters the orbit, passes between the superior and external recti muscles of the eye, and penetrates the external rectus.† He describes also a branch one eighth of a line in thickness, going from the otic ganglion into the spheroidal sinus, another going from it to the vidian, and a third going to the tensor palati muscle.

*Facial nerve.* It was known that in paralysis of the facial nerve of one side the uvula was commonly drawn to the opposite side, and this was supposed to indicate that the facial is the motor nerve of the palate. Some doubt was thrown upon the conclusion by M. Debrou, who showed that the uvula in many persons was naturally not suspended in the middle line. But this objection has been removed by M. Diday,‡ who has observed a case in which the uvula was drawn to the opposite side while the paralysis of one nerve lasted, but gained its straight position when the paralysis ceased. It seems probable, therefore, that at the junction of the superior petrous branch of the vidian with the facial, branches are sent from (not to) the latter which go to the spheno-palatine ganglion, and thence through the posterior palatine nerves to the soft palate, as Soemmering believed.

*Corda tympani.* Dr. Guarini§ states that he can demonstrate visibly that the chorda tympani comes off as a distinct branch from the facial nerve, without any communication with the vidian. He gives the following reasons for believing that through the corda tympani the facial has a motor influence on the tongue. His experiments were often repeated before Panizza and others. 1. When the hypoglossal nerve is galvanized the tongue is moved convulsively forwards and backwards and upwards and downwards, but the fibres of the middle portion are quiet. 2. When the trigeminus is galvanized the tongue never moves. 3. When the facial is galvanized the tongue moves quickly upwards and downwards, and there is a kind of vermiform motion of its middle part. The first movement depends on the styloglossi muscles, which have a distinct branch from the facial; the second on the linguales, to which branches can be traced from each corda tympani. 4. After dividing the hypoglossal nerve the movements of the stytoglossus and lingualis alone continue, and these may be excited by galvanizing the facial. But the vermiform motion does not continue after (in the same case) the chorda tympani is destroyed.

*Nervus vagus* and *nervus accessorius.* Mr. Spence‖ has contributed a fact of great importance to the reconciling of the contrary statements respecting the motor functions of the pharyngeal and inferior laryngeal branches of the vagus nerve. He has traced a filament (distinguished from the rest of the vagus by its white colour,) which, arising from the groove between the olivary and restiform bodies, passes along the course of the vagus trunk, but goes over without joining the superior ganglion, and does not join the vagus trunk till just above the inferior ganglion. At this point of junction it is also joined by the internal branch of the accessorius, and from the junction of the two the pharyngeal branch of the vagus is given off.

* Müller's Archiv, 1842, Heft v.

† An observation of Retzius, made in 1841, that the sixth nerve supplies the retractor muscle of the eye in birds and mammalia, and the muscle of the nictitating membrane in the former appears not generally known by English physiologists, though it is important in the comparative physiology of the eye.

‡ Gazette Médicale, Dec. 24, 1842.

§ Annali Univ. di Medicina, Maggio, 1842, and Schmidt's Jahrbucher, 1843, Heft iii.

‖ Edinburgh Medical and Surgical Journal, Oct. 1842.

The conjoined white cord then descending with the vagus, seems to pass principally into the recurrent nerve, and probably sends filaments into the œsophageal branches.

Mr. Spence, whose dissections agree nearly with those of Bendz, proposes for the separated white cord of the vagus the name of the motor column of the vagus, (motor *root* would perhaps be better,) and likens its arrangement to that of the motor root of the fifth nerve, passing under the Gasserian ganglion, and joining the trunk beyond it. The pharyngeal branch of the vagus and the recurrent laryngeal being thus given off from the internal branch of the accessorius and from a motor root of the vagus, their purely motor functions are sufficiently accounted for; and the experiments of Dr. John Reid, in which irritation of the roots of the vagus nerve produced movements of the larynx, are explained. It is still, however, not clearly explained why his experiments of irritating the accessorius within the skull did not (as those of M. Longet did,) produce movements of the same organ. It is possible that all the filaments of the nerves were not implicated in the irritation.

A confirmation amounting almost to proof of this view of the influence of the nervus accessorius in the movements of the human larynx, is afforded by the fact, that according to Professor W. Vrolik,* the internal branch of the nervus accessorius in the chimpansé does not join the vagus, but goes at once and separately to the larynx, while the external branch is distributed almost exclusively to the trapezius And other facts confirmatory of the same view are supplied by the experiments of Signor Morganti;† which also tend to show that the external branch of the accessorius contains the fibres which have their origin lowest down on the cord, while the internal branch contains those which arise higher up, just below the vagus, of which the author considers the accessorius to be the anterior root.

*Spinal nerves.* Mr. Viner Ellis‡ has described minutely the arrangement of the posterior branches of the spinal nerves. Within the extent of the multifidus spinæ muscle, including therefore all the spinal nerves, except the suboccipital and the last two sacral and the coccygeal, all the posterior divisions of the spinal nerves have an external and internal branch. Of the cervical nerves the external branches supply the cervicalis ascendens, transversalis colli, and trachelo-mastoid muscles; the internal and larger branches supply the multifidus spinæ, semi- and inter-spinales, and those of the four highest give off cutaneous branches. Of the posterior divisions of the dorsal nerves the internal branches penetrate the multifidus spinæ and semispinalis, and give cutaneous nerves from the six upper; the external branches enter the erector spinæ and levatores costarum, and cutaneous portions spring from about the six lower. In the lumbar nerves the internal branches of the posterior divisions end in the multifidus spinæ, and the external branches, giving cutaneous nerves from the three upper, terminate in the erector spinæ. In the three upper sacral nerves the internal branches enter the multifidus spinæ, the external and larger become cutaneous after uniting by anastomotic arches with each other and with the external branch of the last lumbar and fourth sacral nerves.

*Sympathetic nerves.* Dr. A. Von Walther§ has made experiments in which (after exposing them from behind) he has divided upon the front of the spine, near the sacrum and close to the aorta, some four or seven filaments passing from one of the trunks of the sympathetic nerve to the ischiatic plexus. (The mode of separation is accurately described.) The nearly constant result was, that after two days the capillary circulation on the operated side became more rapid, the capillaries smaller, and the blood-corpuscles in them disproportionately few. This lasted till about the fifth day; then for a day the circulation became natural, and then it

* Recherches d'Anatomie Comparée de Chimpansé, p. 40.

† Gazette des Hôpitaux, Août 17, 1843; analysis of an article in the Annali Univ. di Medicina.

‡ Medical Gazette, February 10, 1843. § Müller's Archiv, 1842, Heft v.

became slower and pulsatile, and gradually ceased. The blood-corpuscles accumulated in the larger vessels in spots, exudation took place, and the membrane of the web became soft and rotten.

With one exception in many experiments these changes occurred on only the foot of the injured side; yet, ingenious as they are, there must be much doubt as to the sufficiency of these experiments to prove the influence of these sympathetic nerves upon the irritation of the limb. The mere injury of the other nerves of that side would produce some effect, especially in animals tied down for fourteen or more days.

Dr. Fäsebeck* finds a sublingual ganglion between the mylohoideus muscle and the sublingual gland, about two lines from the lower border of the latter. It is a round, flat, grayish-red swelling, about a line in diameter, and receives branches from the lingual branch of the fifth, from the chorda tympani, and from the filaments of the plexus around the sublingual artery. There proceed from its anterior and inferior part six branches, which penetrate the sublingual gland, and of which one accompanies the duct. He describes also six ganglia, each from one to three lines in diameter, between the lower part of the trachea and œsophagus, and between the latter and the spine, formed on branches of the sympathetic, vagus, and recurrent nerves, and giving filaments to the cardiac plexus, aorta, pulmonary artery, thoracic duct, superior cava, trachea, œsophagus, and pericardium.

### SPECIAL SENSES.

*Eye.* Dr. W. Clay Wallace† has described two new muscles of the eye, to which he has assigned the function of adjusting the focal length of the organ for viewing distant and near objects. They resemble crescents, the horns of which meet at the equator of the eye, and they surround the gray cellular matter connecting the ciliary processes. Their fibres are radiated, and their colour like that of the muscles of the frog's leg. The trunks of the ciliary arteries pass the muscular fibres at the junction of the crescents, and are therefore not affected by their contraction; but the veins pass directly under the muscles and are compressed at each act of contraction. Supposing therefore that the eye is adjusted to a remote object and suddenly directed to a near one, an indistinct image of the latter is formed on the retina. The impression of this image is, Dr. C. Wallace thinks, communicated to the sensorium, and by a reflex impression through the third and fifth (?) nerves the muscles around the ciliary processes are made to contract; the veins of those processes being thus compressed they become erect, and their apices which float in the aqueous humour are elongated; these apices being attached to the anterior wall of the canal of Petit, draw it forward and with it the crystalline lens, till a distinct image of the object is formed on the retina. The return of the crystalline to its place is effected by the relaxation of the muscles, the emptying of the veins, and the elastic retraction of the tissue of the vitreous humour.

The description just given of the muscles is from the eye of the ox: in man they form not two crescents, but an entire ring.

According to Dr. Power,‡ the nervous fibres proceeding from the optic ganglia to the retinæ in the loligo, cross each other's course: those from the back part of the ganglia pass on to the anterior part of the retina, and *vice versâ* the bundles interlacing in the most perfect manner, and like the crossing of the fingers of both hands, passing between one another from one side of the ganglion to the opposite side of the retina. Led by this to the examination of the same nerves in higher animals, he found that a similar arrangement existed in all which he examined; in all, either by interlacement or by a half spiral turn, all the filaments which at its origin are in the upper part of the optic nerve, become near its retinal end inferior, so that the inferior filaments of the retina correspond to superior filaments in the brain.

* Müller's Archiv, 1842, Heft v. † Medical Gazette, Dec. 16 and 23, 1842.

‡ Dublin Journal of Medical Science, January, 1843.

He believes that this arrangement accounts for erect vision, although the impression of an object on the retina must be reversed. And if the observations be true, it is probably not necessary to look further for the explanation of this controverted question.

### GENERATION.

*Testicles.* Mr. Gulliver* has confirmed R. Wagner's observation, that the general enlargement of the testicles which takes place as the period of procreation approaches is accompanied by enlargement of the individual seminal tubes. During winter he finds that the seminal tubes of birds are tolerably thick and strong; but at the season of procreation semen accumulates in them, and their coats are so distended and attenuated that they are very easily ruptured. The same thinning and enlargement of the tubules occurs in the development of the human testicles at puberty.

An interesting case, proving the sympathy of the vital organs with the testicles, is recorded by Dr. Schlesier.† A healthy man engaged in a fray in the dark, was suddenly heard to shriek out: he fell in convulsions and died in five minutes. On examination the only injury found was the rupture of both the spermatic arteries and veins at the internal rings, produced by the scrotum and testicles having been seized and pulled down by one of those with whom the man was fighting.

*Spermatozoa.* Facts of much importance in regard to the formation of spermatozoa are furnished by the cases first recorded by Mr. Liston and Mr. Lloyd,‡ and since repeatedly observed, in which these bodies are found in the fluid of common hydrocele of the tunica vaginalis testis, and in encysted hydrocele.

*Uterus.* In an appendix to his former papers on the nervous system of the uterus, Dr. Lee§ has published a further and very elaborate account of his dissections. The description is not such an one as can be here condensed, but in referring to his original papers, I may be allowed to state, that an examination of the preparations which I have been recently permitted to make, has convinced me that Dr. Lee's account and delineations of them are accurate and complete, and that there can be no reasonable doubt that the structures which he has displayed, are, as he describes them, the nerves and nervous ganglia of the pregnant uterus.

*Ovum—its development, discharge, &c.* The Report of Mr. T. Wharton Jones 'On the Ovum of Man and the Mammifera,' inserted in the last number of this Journal, is so complete to the time of its publication, that little need be now said on this department of physiology. I shall only state at greater length the conclusions recently arrived at respecting the escape of ova independently of fecundation, and the connexion of this occurrence with menstruation, which Mr. Wharton Jones was obliged to compress within the limits of a postscript. Many of these conclusions have been long held on insufficient grounds: those which may now be deemed established are as follows:||

* Proceedings of the Zoological Society, July 26, 1842.

† Casper's Wochenschrift, Oct. 22, 1842.

‡ See their respective papers in the Medico-Chirurgical Transactions, 1843, vol. xxvi.

§ Philosophical Transactions, 1842, part II.

|| The honour of priority in ascertaining many of the facts in these questions has been disputed both here and in France. Being neither willing nor able to decide in such a case, I shall only refer in one note to all the sources in which the recently-adduced facts themselves and the claims of the several candidates for honour may be found:—Dr. Robt. Lee, Lecture in the Medical Gazette, Nov. 4, 1842; Mr. Girdwood, Lancet, March 4, 1843; letters by various contributors, in the following numbers of the same journal; papers by M. Raciborski and Professor Bischoff, in the Comptes Rendus des Seances de l'Académie des Sciences, the Gazette Médicale and l'Expérience, July and August, 1843; Duverney, in the two last-named journals; Pouchet, in the Gazette Médicale, August 19, 1843, in an analysis of his work called Théorie Positive de la Fécondation des Mammifères.

1. Each act of menstruation is connected with the maturation and discharge of an ovum. Numerous cases in proof of this are related (in addition to those formerly recorded by him, and by MM. Gendrin, Negrier, and others,) by Dr. Robert Lee; others by Mr. Girdwood. M. Raciborski has four times found that ova had been recently discharged from the ovaries of virgins who died at or near the period of menstruation; and Bischoff has also four times found Graafian vesicles, containing effused blood, in girls who had recently menstruated.

This menstrual discharge of an ovum is said by Raciborski and Bischoff to be followed by the formation of a corpus luteum, similar to that which is formed when the ovum is impregnated and developed. [But in this I have no doubt they are mistaken. If it were so, one or more corpora lutea should be found in the ovaries of all who die while the habit of menstruation continues; for the *corpus luteum* which forms when impregnation has taken place, is distinct not only through the pregnancy, but for more—often much more—than a month after delivery. Neither are the cavities which are left after the menstrual discharge of ova, or the processes by which they are closed, at all similar to those found when impregnation has taken place. In many examinations of ovaries I have not yet seen a case in which, without impregnation, anything has been found which could be mistaken for a *corpus luteum* formed after an ovum has been discharged and impregnated.] Mr. Girdwood believes that the cicatrices left after the discharge of menstrual ova may be counted, so as to indicate the number of ova discharged and the number of times of menstruation. [But recently I have examined a case in which a girl of seventeen had not menstruated for four months before her death, but previously had menstruated regularly: the ovaries showed no traces of cicatrices. Probably, therefore, the cicatrices remain for a time distinct, but are gradually obliterated, as they are in the nearly analogous case of the discharge of the Peyer's and solitary glands of the intestines.]

3. The menstruation of women, in so far as the periodical maturation and discharge of ova is concerned, is analogous to the *heat* or *rut* of animals. The phenomena, according to Raciborski, may be most distinctly seen in the sow; but in all the domestic mammalia at their period of heat one or more follicles attain their highest degree of development, project upon the surface of the ovary, and at length burst with hemorrhage into their containing cavities, and this whether copulation have taken place or not. Bischoff also has repeatedly found the same things occur in bitches and rabbits whose uterus and tubes have been extirpated: they have heat, the ova mature and detach themselves and pass into the remaining portion of the tube, but of course cannot be impregnated.

4. The discharge of the ova and their passage along the tubes is independent of impregnation and the passage of the seminal corpuscles. This is evident from the facts already mentioned; and others are furnished by Bischoff. In one experiment he kept a bitch carefully secluded till the period of heat ensued. She then copulated once, and immediately after he extirpated the left uterine horn, ovary, and oviduct. The copulation had lasted a quarter of an hour; and he found that the semen had penetrated to the upper angle of the uterine horn, but not into the tube. He found also five ova in the oviduct more than two inches from its abdominal orifice; a distance sufficiently great to prove that they had not been detached in the copulation. Next day he killed the bitch, and found that spermatozoa had reached about a quarter of an inch in the right tube; he found also five ova in the same tube, and as many *corpora lutea* in the right ovary, but none of the spermatozoa had come in contact with the ova. These cases proved the detachment of ova before copulation. In some others Bischoff found that they were not detached till long after the act. In some he found that they were undetached twenty-four hours after copulation, and that the seminal corpuscles had passed on towards them. In others also he found the independence of the passages of the ova and the semen still more marked; for example, several days after copulation, ova were found fecundated in one tube, but in the other spermatozoa alone, none of the Graafian vesicles in the corresponding ovary being either enlarged or fully developed.*

5. Thus, according to the period of heat at which copulation takes place, will be the place at which the semen meets the ovum. If it be early, the ovum may not escape before the semen reaches the ovary; if late, the ovum may have arrived at the uterus; and probably if it have arrived at the lower or uterine third of the tube before it comes in contact with the semen, impregnation is impossible on account of the changes which the vitellus has already undergone. In women it is in like manner near the period of menstruation that impregnation is most likely to occur. It may take place just before menstruation if the ovum be just mature when the semen reaches the ovary; or some days after, the ovum after its discharge remaining impregnable till the semen reaches it. Or, again, as many analogous circumstances in lower animals prove, an ovum may by the sexual excitement be hurried on to its muturity and discharged; and so, in unusual cases, impregnation may take place at a greater than usual distance from the menstrual period. Still the most common time must be, as common observation shows it is, either during or very near the menstrual period. M. Raciborski has found that in one hundred women there are not more than six or seven in whom this is not the constant rule.

6. All these circumstances prove a closer analogy than was supposed to exist between the discharge of the ova of mammalia and those of the fish, batrachia, and others in which the ova are discharged from the body and impregnated external to it. In all alike the discharge of the ova is an independent act; the differences are in the distances from the ovaries at which the semen is usually brought into contact with it.

Dr. J. E Panck† has published an essay on a case in which he believes that he has made the discovery of the organic connexion between the fallopian tube and the ovary of the human female soon after connexion. A girl, twenty-three years old, was suffocated by carbonic acid five days (it was supposed) after her first conception. There were signs of turgescence about all the uterine and ovarian vessels; the uterus itself was large and vascular, and thickly lined by mucus like a decidua and by ciliary epithelium-cells. On the right side the fallopian tube was turned backwards, and its fringe was expanded over the ovary. They were held together by a fine transparent membrane, which extended over them, over the posterior surface of the uterus and right broad ligament, and a little over the left broad ligament, and was slightly adherent to them all. The left tube and ovary were natural; the right ovary was large and vascular. Directly below the attached fimbriæ there was a cavity in the right ovary, like a distended Graafian vesicle, covered only by serous membrane, about three lines in diameter, and containing a blackish substance like clotted blood. But neither in this nor any where else was an ovum found; so that the evidence of the case is far from complete.

*Formation and structure of the membranes, &c.* M. Serres,‡ in a paper read

* These facts bear on the question of the possibility of a woman conceiving by two different men; and I find a recent notice of a case, often referred to, of a negress who having, as it was believed by herself and others, conceived twice in the same night, first by a negro and afterwards by a European, bore twins, of which one was a pure negress the other a mulatto. Dr. Hille, a Dutch military surgeon in Surinam, where the delivery occurred, adds that the children were living in 1841, that they were eight years old, that the black child, which was at first the strongest of the two, remained so, and that the mother had died some time previously, and on examination was found to have normally formed genital organs. (Casper's Wochenschrift, Jan. 28, 1842.)

† A full account of the supposed discovery is in Casper's Wochenschrift, Mai 27, 1843.

‡ See the Gazette Médicale and contemporary French journals, Juin et Julliet, 1843. MM. Maignien and Jacquart have since published a case (Gazette Médicale, Novembre 4, 1843,) which confirms, they think, M. Serres' view of the amnios. They found in an early aborted embryo an amniotic vesicle fixed to the chorion by a narrow pedicle, near before the Institute of France, and in subsequent discussions, has maintained the view of Pockels, that the embryo is outside the amnios to the fifteenth or twentieth day, and that the amnios up to this time is a free vesicle, in which the embryo dips and envelopes itself (exactly as the ovum is supposed to envelope itself in the decidua) in a double sac. He adds, further, his belief that the allantois of the human embryo, having its pedicle immediately in front of the caudal prolongation, and at a distance from that of the umbilical vesicle, cannot be regarded as produced by a retroversion of the intestine, but has its origin in the corpora Wolffiana, whose existence in the human embryo he considers he has fully demonstrated. His view was supported by preparations, but in the discussions which followed the reading of the memoir, MM. Coste and Velpeau maintained each his own previous view of the matter, and said that the preparations did not demonstrate that of M. Serres.

Mr. John Dalrymple* has described and figured the minute vessels of the vitelline membrane and allantois of the chick. Of the vitelline membrane he says that immediately around the remains of the vitelline area are seen on the internal surface of the yelk-sac the commencement of a series of radiating folds, which as they advance dip deeper and deeper into the interior of the sac, and separate more widely from each other. When the vitelline cells are completely removed it is seen that vessels alone constitute the framework of these folds, the large trunks forming their bases, while innumerable lesser branches dip deep into the interior of the sac, inosculating repeatedly, and terminating in a series of very tortuous branches, which fringe the extreme edge of each fold. Numerous simple loops are observed shooting from the sides of the larger trunks; and if we conceive each trunk and every small vessel thickly covered with an aggregated arrangement of vitelline globules or nucleated cells, which conceal the vessels and colour them bright yellow, we shall have a true idea of the appearance of these folds previous to the manipulation necessary to display the injection.

In the allantois Mr. Dalrymple says there is a very minute distribution of equal-sized capillary vessels throughout its inner layer, forming an uniform vascular surface covering the large trunks as well as the interspaces of their divisions; and the anastomoses of the capillaries are so numerous and close that the areas they leave do not exceed the diameter of the vessels themselves. From the similarity of this arrangement of vessels to that found in the lungs of the frog, salamander, &c., he thinks evidence may be adduced for the supposed respiratory function of the allantois.

Mr. F. Renaud,† confirming (as nearly all now do) E. H. Weber's description of the arrangement of the vessels of the placenta, points out as a chief source of fallacy in the examination of these structures, the rapidity with which the villi of the chorion absorb water, and are distended and confused by it.

M. Elsaesser‡ has found in 144 fœtuses either born dead or living only a month, that in fifty-two born dead (of which forty-eight were mature and four immature), the ductus arteriosus, ductus venosus, and foramen ovale were all open forty-eight times. In four (one immature) the for. ovale was closed, the others open.

In ninety-two dying in the first four weeks (of which twenty-two were premature) all the passages were open in fifty-eight. In eighty the foramen ovale was open; in seventy-seven the ductus arteriosus; in sixty-five the ductus venosus.

The most common mode of closure is: 1. The ductus venosus, beginning at which was the embryo, rather more than a line in length, free at its cephalic extremity, and adhering to the amnios only by its caudal extremity and the inferior part of its dorsal surface.

* Transactions of the Microscopical Society of London, vol. i, 1842.

† London and Edinburgh Monthly Journal of Medical Science, March, 1843.

‡ Henke's Zeitschrift, t. xlii, and Archives Gén. de Médecine, Juillet, 1843. In a later paper (Henke's Zeitschrift, B. iv., No. 42,) M. Elsaesser has given accurately the lengths and weight of 1000 children born at the full period; but in such a form, that no abstract is possible.

the vena portæ. 2. The ductus arteriosus beginning at the middle. 3. The foramen ovale by the application of its edges. Even later than four weeks any of them may sometimes be found partially open.

### LACTATION.

M. Mandl* confirms the view of Henle and others in regard to the perfect milk-corpuscles, proving the existence of an external membranous envelope by rubbing the corpuscles between glasses. The oil-globules are set free, and the torn membranes are unrolled and flattened.

M. Raciborski† has examined the question of the influence of menstruation on the secretion of milk, and has found that it is unimportant. The only difference between the milk of nurses who do, and those who do not menstruate, is that in the former the proportion of cream is rather less in the menstrual period than it is in themselves in the intervals, and than it is generally in non-menstruating nurses.

### PHYSICAL HISTORY OF MAN.

*Characters of the Egyptian and Negro races.* Dr. S. G. Morton‡ has made observations on one hundred crania of ancient Egyptians, obtained at seven sepulchral localities from Memphis in Lower Egypt to Deboud in Nubia. He classes them as 1. *Arcto-Egyptians,* including the purer Caucasian nations, as seen in the Semitic tribes of Western Asia, and the Pelasgic of Southern Europe. 2. Austro-Egyptians, in which the cranium blends the characters of the Hindoo and Southern Arab; which people, the author thinks were ingrafted on the original population of Ethiopia, and thus gave rise to the celebrated Meroite nations of antiquity. 3. Negroloid, in which the osteology of the crania corresponds to the Negro; but the hair, though harsh, is long and smooth, like the present Mulatto grades. 4. Negro.

The lines between these could not be exactly drawn. But in the one hundred skulls there might be reckoned fifty-six Arcto-Egyptians, twenty-eight Austro-Egyptians, six Semetic, seven Negroloid, one Negro, and two doubtful.

He deduces, therefore, 1. That Egypt was originally peopled by the Caucasian race. 2. That the great preponderance of heads like those of the purer Caucasians suggests that the valley of the Nile derived its primitive inhabitants from one of these sources. 3. That the Austral-Egyptian or Meroite communities were in great measure derived from the Indo-Arabian stock; thus pointing to a triple Caucasian source for the origin of the Egyptians, when regarded as *one people* extending from Meroe to the Delta. 4. That the Negro race exists in the catacombs in the mixed or Negroloid character: that even in this modified type their presence is comparatively unfrequent; and that if Negroes, as is more than probable, were numerous in Egypt, their social position was chiefly in ancient times what it now is,§ that of plebeians, servants, and slaves.

*Stature.* Some very interesting observations on the stature of man have been made by Mr. A. Shaw.|| He shows that rickets not only produces softening of the bones but arrests growth; and this in the lower extremities much more than the upper, so that the child-like form, characterized by largeness of the head, trunk, and upper extremities is persistent. These three parts are in persons stunted by rickets reduced only by 1-13 of the natural size; but the pelvis and lower extremities are reduced by 1-3. There is, therefore, an *arrest of development* in regard to proportion of form.

This is shown further in that the proportion of size between the cranium and face remains as in childhood; the former being always *proportionally* large, though

* Anatomie Microscopique, livre ix, and Bulletin de l'Académie Royale de Médecine, 1842, p. 1157.

† Bulletin de l'Académie Royale de Médecine, Juin 15, 1843.

‡ On the Form of the Head, and other Ethnographic Characters of the Ancient Egyptians; in the Proceedings of the American Philosophical Society, Nov. 1842.

§ In America.

|| Medico-Chirurg. Trans. vol. xxvi, p. 336.

*absolutely* not so large as in the well-formed adult. The proportions are in the child as 8 :1 ; in the well-formed adult as 6:1 ; in the rickety adult as $7\frac{1}{13}$ :1.

On the other hand, where growth is preternaturally active, as in giants, the lower half of the body is the part which is most increased, and it acquires disproportionate length. And in these, the cranium, though absolutely large, is, relatively to the face, small ; e. g., in the skull of O'Byrne, the giant eight feet high, in the museum of the Royal College of Surgeons, the proportion of the size of the head to that of the face is only as five to one.

*Varieties of the pelvis.* Dr. Knox,* in his "Contributions to Anatomy and Physiology," shows that many or all the national peculiarities of the form of the female pelvis, as well as many of those which are regarded as malformations, are to be regarded as due to the fœtal form of the pelvis being more or less persistent. The fœtal form "is more quadrilateral than oval or rounded, and its antero-posterior diameter is the longest: it has the form, in great measure, of the pelvis of the quadruped and quadrumanous mammal, of the human male generally, and of certain ill-formed human female pelves." When the persistence in this form exists on one side only of the pelvis, it produces the *pelvis obliquè ovata* of Naegele. Its more common effect when existing on both sides is to produce the not unfrequent narrow quadrilateral form of female pelvis ; but when it exists in an extreme degree on both sides, it may produce, as in a pelvis in Dr. Outrepont's collection, a kind of a double Naegele's oblique pelvis—one with a very long conjugate diameter, but very narrow in front—almost like a seal's pelvis.

He gives cases also of relaxation of the ligaments of the symphysis pubis in delivery.

*Age of puberty in girls.* Mr. Roberton† of Manchester, in continuation of some former papers, the object of which was to prove that the age of puberty is as early in the cold as in the tropical regions of the earth, and that the early fecundity in Hindostan and other warm countries is only the consequence of early marriages, proceeds now to show, that in all countries alike, early marriages (and early fecundity) are always connected with moral and political degradation, as exhibited in bad laws and customs, the enslavement more or less of the women, ignorance of letters, impure and debasing systems of religion ; and that they bear no relation to the climate of the country.

His evidence is extensive and very interesting ; and the conclusions he arrives at are, 1. That in England, Germany, and Protestant Europe in general, early marriage, i. e. marriage about the age of puberty, is comparatively rare. 2. That early marriage prevails among the uncivilized tribes within the arctic circle, as it likewise does in *all* cold countries, the inhabitants of which are in a state of ignorance and moral degradation. 3. That throughout European Russia, which is confessedly low in civilization, extremely premature marriage was the universal custom at no distant date. 4. That at the present day, in the most southerly countries of Europe, where the people are immersed in superstition and ignorance, marriage is early. 5. That in Ireland, which as to its moral condition somewhat resembles the last-mentioned countries, the marriage union takes place among the Roman Catholic population almost as early. 6. That in England, about two centuries ago, when debasing political and social circumstances combined to favour the practice, early marriages were general, at all events in the upper ranks. 7. That in all the countries to which reference has been made, juvenile marriage is invariably seen as an attendant upon ignorance and moral debasement, and this without reference to *climate.* 8. That consequently it is allowable to infer that early marriage in oriental countries (which has generally, but without any proof, been ascribed to precocious puberty,) depends solely on the same moral and political causes as produce it elsewhere ; more especially as those very causes are well known to exist at present in an aggravated degree in all oriental and intertropical countries.

* Medical Gazette, July 21 and following numbers, 1843.

† Edinburgh Medical and Surgical Journal, Oct. 1832 and July 1842.

These conclusions are probably in a great measure true; yet that the commencement of menstruation and of fecundity does bear some relation to the latitude and average temperature, appears to be proved by the following table, in which M. Raciborski gives his results as to the average age at which menstruation commences in different countries and towns:

| Name of Town. | Latitude. | Age at first menstruation. | Mid. Temp. of the year. | No. of Observations. | Observer. |
|---|---|---|---|---|---|
| *Toulon ......... | 43° ... | 14·081 ... | 15·° ... | 43 ... | Marc d'Espéne. |
| †Marseilles ...... | 43 .. | 14·015 ... | 15· ... | 25 ... | Ditto |
| †Lyons ............ | 46 ... | 14·492 ... | 11·6 ... | 160 ... | Bouchacourt. |
| Paris ............ | 49 ... | 14·465 ... | 10·6 ... | 200 ... | Raciborski. |
| Göttingen ...... | 52 ... | 16·038 ... | 8· ... | 137 ... | Osiander. |
| Warsaw ......... | 52 ... | 15·083 ... | 9·2 ... | 100 ... | Lebrun. |
| ‡Manchester ...... | 53 ... | 15·191 ... | 9·6 ... | 450 ... | Roberton. |
| Skeen ............ | 59 ... | 15·450 ... | 6· ... | 100 ... | Faye. |
| Stockholm ...... | 59 ... | 15·590 ... | 5·7 ... | 102 ... | Wistrand. |
| §Swedish Lapland | 65 ... | 18· ... | 4· ... | ... | Wretholm. |

In general, therefore, the period of puberty is later in nearly the same ratio as the latitude is higher: for each degree of the one the other is retarded about a month and a few days. And the lower the latitude, the more frequent are the examples of precocious appearance of menstruation.

A still more exact relation is between the date of first menstruation and the mean year's temperature; as may be seen by comparing Warsaw and Göttingen, Göttingen and Manchester, &c. M. Raciborski adds that *race* often determines the period of first menstruation. The children of negroes born in England menstruate as early as their parents; those of Europeans born in India as late as their parents. To determine how far circumstances of climate could countervail the influence of race, M. Raciborski obtained information respecting the period of menstruation in Jewesses in Poland, from M. Lebrun, médecin-en-chef of a hospital in Warsaw, and found the mean period in Catholics 15·83, in Jewesses 15·89; (100 observations of each race;) showing that the influence of race remained after ten or more centuries. And in like manner the menstruation ceases sooner in Polish Jewesses than in Sclavonian women, lasting in the former on an average $29\frac{28}{33}$ years, and in the latter $31\frac{6}{33}$ years.

There is a difference also, dependent, probably, on numerous causes, between the women of Paris and those of villages a league and half or two leagues from Paris, though both have a similar soil, temperature, &c. In the villages the average age at first menstruating is 15·020 years, in Paris 14·465.||

M. Raciborski¶ has also published an account of the age at which menstruation ceases. At Lyons the average age is between 45 and 50; at the Slp êtrière, in 100 women, the average was 46·03: at Warsaw, 47·05; at Christiana, 48·07. As a general rule, the greater the number of children borne, the longer is the continuation of menstruation; and the earlier the commencement of menstruation, the greater the number of children and the later the cessation.

*Changes in age—Varia.* A remarkable example of vigour in most advanced age has been observed in M. Rochard, a musician, 107 years old, on whom Mr. Cæsar Hawkins successfully operated for strangulated hernia.** The hernia had

* Archiv. Gén. de Méd. 1835.

† Dict. des Sc. Méd. 2me edit. 'Menstruation.'

‡ Edinb. Med. and Surg. Journ. Oct. 1832.

§ Eighteen years is only a general statement, it should probably be less.

|| A. Raciborski 'De l'Epoque de la Pubèrté,' &c., L'Expérience, Juillet 26, 1843, and many subsequent numbers. Numerous facts bearing on this and similar questions may be found in Brierre de Boismont, 'De la Menstruation,' &c., Paris, 1842, reviewed in vol. XIV, Oct. 1842.

¶ Medical Gazette, Dec. 9, 1842.

** L'Expérience, Octobre 26, 1843.

been strangulated upwards of thirty hours: the wound united by the first intention, except where two ligatures hung out, and in a fortnight after the operation the old man was as well as before it. He has since died.

M. Ruelle,* of Cambrai, has recorded an example of precocious virility. A child, three years and a quarter old, muscular and strong as one of eight, has all his male organs of the full adult size, with long black hair on the pubes, and under excitement discharges semen four or five times daily. He has also a full male voice, and dark short hair on the cheek and upper lip.

A contribution to the knowledge of the effects of the air at great heights in the atmosphere, and a confirmation of most of the results which have been already obtained are furnished in the observations made in an account of an ascent of the 'Grosse Venediger,' a mountain upwards of 11,000 Austrian feet high, in the southern border of Oberpinzgau, by Dr. F. Spitaler † The party ascending consisted of forty persons, of whom twenty-six only accomplished the feat. The chief effects produced were, 1. On the respiration, which in all became rapid and difficult, and was greatly hurried in exertion, and in some amounted to agony, so that from mere dyspnœa they were compelled to return: one also had slight hemoptysis. 2 On the pulse, which became small and weak. 3 On the secretion of urine, which was remarkably diminished, so that among the whole forty persons, during between eight and nine hours' walking in a temperature only just above freezing point, urine was passed only nine times. 4. On the cutaneous exhalation, which (though the invisible evaporation was probably much increased,) did not once appear as sweat. 5. On the heat of the body. All had the sensation of intolerable cold, though actively exerting themselves and well clothed, and though the temperature was 4° or 5° R, and the weather nearly calm. This was felt, however, only when they had ascended above 9000 feet: below it, although the temperature was not higher, the sensation of cold was far less painful. 6. On the muscular power, which was, in all the party after they had nearly attained the height of 1000 feet, exceedingly prostrated. Some were from utter fatigue obliged to give in; some could not even stand, and of those who went on none could walk more than twenty or, at last, more than ten steps without stopping to rest. In some these signs were accompanied by ringing in the ears, in some by nausea and even by vomiting, in some by utter carelessness of life; not one reached the summit except in a state of complete exhaustion. And all this was far from being the kind of fatigue consequent on extraordinary muscular exertion; for several of those who could not attain the summit and of those who did so only with difficulty descended in good plight and walked on for many hours with scarcely a complaint of weariness.

The influence of the increased brightness of the sun's light was very marked. The clear, deep blue of the most beautiful southern sky was far surpassed in beauty; and though all the party wore dark shades or veils, all suffered from pain and inflammation of the face and eyes and of every part which was at all exposed to the action of the sun.

Dr. D. D. Owen‡ has given a detailed account of the impressions of two human feet found on the surface of a slab of limestone, from the specimen described by Mr. Schoolcraft in 1822, and by Dr. Mantell in his 'Wonders of Geology.' The slab of limestone was taken from a rock which was exposed at the very margin of the Mississippi, opposite St. Louis, but only during very low water, such as does not happen more than once in ten years. It is a solid mass, upwards of a ton weight, of a purple and grayish tint, containing numerous shells, species of producta and pentromytes The impressions are so exactly like those of feet set upon a soft mud, that it is difficult to imagine that they can be a work of art; yet Dr. Owen thinks the difficulties of this hypothesis less than those of that which ascribes to them an existence before the limestone had hardened.

* Bulletin de l'Académie de Médecine, Févr. 28, 1843.

† Oesterreichische Med. Jahrbucher, Oct. 1843. A summary of nearly all the observations on this subject is given by M. Rey, in the Revue Médicale, Oct. 1842.

‡ American Journal of Science and Arts, July, 1842.

The contents of the following papers have considerable interest, but are not of a kind which could be introduced into the Report:

Mr. Sibson's observations "On the relative positions of the thoracic and abdominal viscera," of which an abstract is given in the Lancet of August 12, 1843, and the Provincial Medical and Surgical Journal of the same date. Dr. Laycock's papers "In proof of the existence of a general law of periodicity in the phenomena of life," in the Lancet, October 22, &c., 1842. Mr. Ancell's "Commentaries on the works of Liebig," Lancet, November 13, &c., 1842. Papers by M. J. Parise in the Archives Générale de la Médecine, Juillet et Août, 1843, "On the apparent changes in the length of the lower extremities in changes of their position in regard to the pelvis." Numerous papers "On comparative and transcendental anatomy," by Dr. Knox, in the Medical Gazette. Dr. Willis's papers on the "Physiology of the skin and the lymphatics," in the Proceedings of the Royal Society March 9, 1843, and in the contemporary journals. Dr. Golding Bird's paper "On the microscopic globules found in urine," in the Guy's Hospital Reports. Mr. Gulliver's observations "On the frequency of fatty deposits in the degenerations of the tissues of old persons," in the Medico-Chirurgical Transactions, vol. xxvi. J. von Berres, Erfahrungen über die Zeugung, in the Oesterreichische Jahrbucher, April to September, 1843.

